# Targeting the Achilles Heel of FtsZ: The Interdomain Cleft

**DOI:** 10.3389/fmicb.2021.732796

**Published:** 2021-09-08

**Authors:** Pinkilata Pradhan, William Margolin, Tushar Kant Beuria

**Affiliations:** ^1^Institute of Life Sciences, Nalco Square, Bhubaneswar, India; ^2^Regional Centre for Biotechnology, Faridabad, India; ^3^Department of Microbiology and Molecular Genetics, McGovern Medical School, Houston, TX, United States

**Keywords:** protein structure, tubulin, bacterial cell division, small molecule inhibitor, antibacterial, ftsZ

## Abstract

Widespread antimicrobial resistance among bacterial pathogens is a serious threat to public health. Thus, identification of new targets and development of new antibacterial agents are urgently needed. Although cell division is a major driver of bacterial colonization and pathogenesis, its targeting with antibacterial compounds is still in its infancy. FtsZ, a bacterial cytoskeletal homolog of eukaryotic tubulin, plays a highly conserved and foundational role in cell division and has been the primary focus of research on small molecule cell division inhibitors. FtsZ contains two drug-binding pockets: the GTP binding site situated at the interface between polymeric subunits, and the inter-domain cleft (IDC), located between the N-terminal and C-terminal segments of the core globular domain of FtsZ. The majority of anti-FtsZ molecules bind to the IDC. Compounds that bind instead to the GTP binding site are much less useful as potential antimicrobial therapeutics because they are often cytotoxic to mammalian cells, due to the high sequence similarity between the GTP binding sites of FtsZ and tubulin. Fortunately, the IDC has much less sequence and structural similarity with tubulin, making it a better potential target for drugs that are less toxic to humans. Over the last decade, a large number of natural and synthetic IDC inhibitors have been identified. Here we outline the molecular structure of IDC in detail and discuss how it has become a crucial target for broad spectrum and species-specific antibacterial agents. We also outline the drugs that bind to the IDC and their modes of action.

## Introduction

The battle against infectious diseases has been a persistent challenge for humans. The development and use of antibiotics helped to prevent and control bacterial infections, but at the same time its misuse led to the development of antibacterial resistance (Ma and Ma, 2012). An increase in antibacterial resistance is now of significant concern worldwide, resulting in higher infection and mortality rates. As more bacteria become resistant to currently available antibiotics, discovery of new antibiotics and identification of new targets is more urgent than ever.

Although division of bacterial cells is key for their colonization and pathogenesis, the cell division machinery has not been fully explored for the development of antibacterial agents despite many breakthroughs in the mechanisms and regulation of this fundamental process. Cell division is initiated by the formation of a discontinuous and dynamic circumferential assembly at the site of division called the Z ring, which is located at the cell midpoint in bacteria that divide by binary fission.

Several proteins are involved in determining the proper assembly and correct placement of the Z-ring (Hale and de Boer, 1997; Pichoff and Lutkenhaus, 2002; Bramkamp et al., 2008; Rowlett and Margolin, 2015). However, the key organizing protein is FtsZ (**F**ilamenting **t**emperature **s**ensitive mutant **Z**), which assembles into treadmilling polymers to form a dynamic skeleton for the Z-ring, ultimately recruiting other cell division proteins to the Z-ring in a sequential manner (Bi and Lutkenhaus, 1991; Wang et al., 2020). FtsZ is present in nearly all bacteria, plant plastids, and many archaea, and is a homolog of eukaryotic tubulin (Mukherjee and Lutkenhaus, 1994; Erickson, 1995; de Pereda et al., 1996; Nogales et al., 1998a, b; Kaur et al., 2010). In search for new antibiotic targets, FtsZ has become the leading candidate, as it is essential for cell division in most bacteria and is absent in eukaryotes (Beall and Lutkenhaus, 1991; Dai and Lutkenhaus, 1991; Pinho and Errington, 2003; Li and Ma, 2015). Although FtsZ is homologous to eukaryotic tubulin, it shares little sequence identity (10–18%) with tubulin, reducing the likelihood that drugs targeting FtsZ will be toxic to eukaryotic cells (de Pereda et al., 1996).

Over the past few decades, researchers have characterized several natural as well as synthetic FtsZ inhibitors. However, the interaction sites/binding pockets in FtsZ for many inhibitors are not yet fully characterized. To define the functional groups in a small molecule that can efficiently affect the functions of a target, it is critical to understand its binding site in the target. A detailed molecular understanding of the binding site will help to design and develop specific drugs against the target, which will further help to identify more specific and potent FtsZ inhibitors.

FtsZ contains two prominent drug binding sites, the GTP binding site, which we will refer to as the nucleotide binding domain (NBD), and the inter-domain cleft (IDC) (Casiraghi et al., 2020). The NBD is similar to that of tubulin and shares the glycine-rich signature motif GGGTG(T/S)G of tubulin (de Pereda et al., 1996; Löwe, 1998). Consequently, there is a higher chance that drugs that target the FtsZ GTP binding site may also interact with tubulin and cause toxicity in the mammalian cells. In contrast, the IDC of FtsZ exhibits less similarity to tubulin, reducing the odds of toxicity to mammalian cells (Casiraghi et al., 2020). Fortunately, most of the reported FtsZ inhibitors interact with the IDC. This review highlights different drugs that target the IDC, summarizes the residues within the IDC that are important for drug binding, and outlines what is known about the mechanism of action. We also describe why the FtsZ IDC has attracted more attention as a drug target for the development of novel antibacterial compounds.

## FtsZ and the Z-Ring

Bacterial cell division is a complex process that involves replication and segregation of its genetic material, elongation of the lateral cell wall, and formation of a division septum at midcell followed by separation of the two daughter cells. Using immune electron microscopy on *Escherichia coli* cells undergoing binary fission, Bi and Lutkenhaus provided initial proof 30 years ago that FtsZ localizes at the center of the cell and forms a ring like structure (Bi and Lutkenhaus, 1991). The correct localization of the Z-ring at midcell in many rod-shaped bacteria is controlled by diverse spatial regulatory systems. In *E. coli*, the nucleoid occlusion system prevents potentially DNA-damaging formation of the Z-ring over the unsegregated nucleoid, while the Min system oscillates between both cell poles and inhibits the formation of Z-rings at the poles (Rowlett and Margolin, 2015; Taviti and Beuria, 2017). The invagination of the cell envelope behind the Z-ring at midcell is initiated by forces generated by the Z-ring. Several independent studies showed that a ∼8 – 80 pN force generated during the constriction of the Z-ring may be sufficient to initiate this invagination (Lan et al., 2007; Hsin et al., 2012; Yao et al., 2017; Nguyen et al., 2019; Ramirez-Diaz et al., 2021).

Assembly of the Z-ring is regulated by a number of endogenous activator and inhibitor proteins, maintaining a balance between instability and stability (Hale and de Boer, 1997; Trusca et al., 1998; Pichoff and Lutkenhaus, 2002; Haeusser et al., 2004). Overproduction of FtsZ inhibitors such as SulA or MinCD, or inactivation of FtsZ stabilizing proteins such as FtsA, ZipA, or Zap proteins in *E. coli* lead to long, filamentous cells without Z-rings or with multiple stalled Z-rings (Addinall et al., 1996), ultimately preventing viability. Similarly, small molecules that inhibit Z ring formation or hyperstabilize the Z-ring also lead to a block in cell division. As FtsZ is the most important component of the Z-ring, development of FtsZ inhibitors requires a molecular understanding of FtsZ structure, drug-binding sites on FtsZ and the inhibitory effects of drugs on FtsZ functions.

## Overall FtsZ Structure and Function

### Domains of FtsZ

**FIGURE 1:**
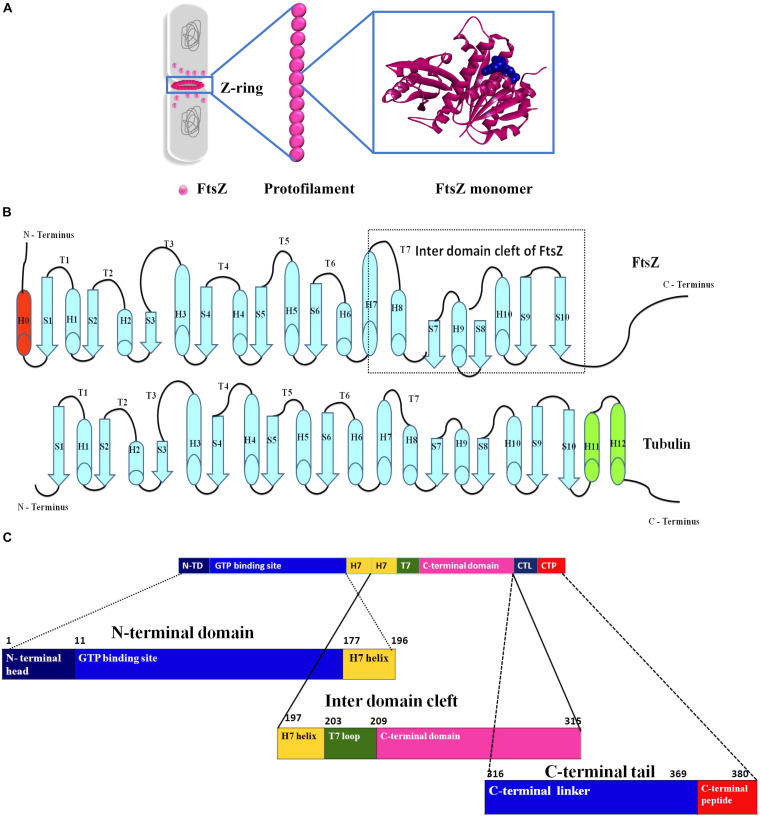
Insight into the structure of FtsZ. **(A)** During bacterial cell division the nucleoids (gray) are segregated and a Z-ring (magenta) is formed at midcell by the coalescence and assembly of FtsZ monomers from the cytoplasm into polymers. Monomers are continuously exchanged with treadmilling FtsZ polymers. **(B)** Secondary structures of both FtsZ and tubulin. **(C)** An outline of the domains of FtsZ.

FtsZ consists of a conserved globular core (residues 10–316) comprising an N-terminal domain (H1–H6, S1–S6), a core helix (H7), a spacer loop (T7 loop) and a C-terminal domain (H8–H10, S7–S10), which in turn is connected to a conserved peptide at the extreme C terminus (residues 369–383) by a flexible unstructured linker (317–368) (de Boer et al., 1992; Löwe and Amos, 1998; Nogales et al., 1998b) (Figure 1). In some species, such as the archaeon *Methanocaldococcus jannaschii*, FtsZ contains an additional helix (H0) in its N-terminal subdomain (Löwe, 1998).

In *E. coli* FtsZ, the N-terminal domain spans residues 1–177. It contains an unstructured and poorly conserved extreme N terminus and a highly conserved NBD (Löwe and Amos, 1998; Vaughan et al., 2004; Gardner et al., 2013). Although the N-terminal domain contains the NDB, it is not sufficient for hydrolyzing GTP (Jindal and Panda, 2013). In *E. coli*, H7 extends from residues 178 to 201, connects the N-terminal and C-terminal domains of the core, and acts like a sliding door for the opening and closing of the IDC. Some residues of H7 are crucial for FtsZ assembly. For example, a single mutation in *Bacillus subtilis* FtsZ (BsFtsZ) R191 can impede FtsZ assembly (Dhaked et al., 2016).

The highly conserved T7 loop in *E. coli* FtsZ (residues 202 – 209) connects H7 to H8 of the C-terminal subdomain and contains a conserved GXXNXD sequence that is important for GTP hydrolysis. Upon FtsZ assembly, the T7 loop of one FtsZ monomer inserts into the GTP binding pocket of the adjacent FtsZ monomer and initiates GTP hydrolysis (Löwe and Amos, 1999; Scheffers et al., 2002). Drug molecules that bind to this site affect GTPase activity of FtsZ.

The C-terminal domain of the globular core of FtsZ (residues 210 to 316) is highly conserved both in sequence and structure. It consists of helices H8–H10 and beta sheets S7–S10. H10 is notably rich in acidic residues and interacts with Min proteins (Taviti and Beuria, 2017). This domain is followed by an unstructured C-terminal linker (CTL) that is highly variable both in composition and length (Taviti and Beuria, 2017). In *E. coli*, the CTL is ∼ 52 residues (317 – 369). In most FtsZs, the CTL connects the globular core domain of FtsZ with a highly conserved 10–20 residue peptide at the extreme end of the C-terminus called the C-terminal peptide (CTP) (Cohan et al., 2020). Although this peptide (residues 369–379 in *E. coli)* is not required for FtsZ assembly, it is crucial for interactions with other membrane-associated cell division proteins like ZipA and FtsA (Ma and Margolin, 1999; Ortiz et al., 2016). Residues D373, I374, F377 and L378 of *E. coli* FtsZ are specifically involved in these protein-protein interactions (Buske and Levin, 2012). As a result, deletion of the CTP blocks FtsZ functions and bacterial division (Din et al., 1998).

### FtsZ Assembly and GTPase Activity

FtsZ, in the presence of GTP, polymerizes into head-to-tail protofilaments (Bramhill and Thompson, 1994; Mukherjee and Lutkenhaus, 1994), which then coalesce to form the Z-ring (Haeusser and Margolin, 2016). The Z–ring is anchored to the membrane with the help of other essential cell division proteins, such as FtsA and ZipA of *E. coli* (Pichoff and Lutkenhaus, 2002). In Gram positive bacteria as well as FtsZ-containing archaea, SepF is the key membrane anchor for FtsZ (Duman et al., 2013; Nussbaum et al., 2021; White and Eswara, 2021).

*In vitro* studies suggest that FtsZ assembles into short protofilaments made up of ∼30 subunits that combine laterally to form the Z-ring (Erickson et al., 2010). These lateral interactions between FtsZ protofilaments help to drive division septum formation (Krupka and Margolin, 2018; Squyres et al., 2021; Whitley et al., 2021). A single FtsZ protofilament is ∼5 nm thick with slightly curved morphology, which becomes highly curved upon GTP hydrolysis (Lu et al., 2000; Romberg et al., 2001). One model proposed that GTP hydrolysis provides the required force for Z-ring constriction and septation (Allard and Cytrynbaum, 2009). As GTP hydrolysis is induced upon FtsZ assembly into polymers, FtsZ subunits within the Z ring are highly dynamic, with a half time of FtsZ subunit turnover as low as 8–9 s in *E. coli* and *B. subtilis* (Anderson et al., 2004). This turnover results from treadmilling, which allows FtsZ polymers to travel circumferentially around the site of septum formation by loss of subunits at one polymer end and gain of subunits at the other (Bisson-Filho et al., 2017; Yang et al., 2017). Surprisingly, only about ∼30% of the FtsZ in *E. coli* cells is actually in the Z-ring at any one time, while the remaining FtsZ circulates in a cytoplasmic pool that is continuously exchanged with treadmilling FtsZ polymers that comprise the Z ring (Stricker et al., 2002). Despite the rapid turnover observed in cells, purified FtsZ in solution hydrolyzes its bound GTP at a rate of only ∼2 GTP per FtsZ molecule per minute (Lu et al., 1998), suggesting that cellular factors may enhance FtsZ GTPase activity. Molecular dynamics simulations of FtsZ dimers predict the forces generated by GTP hydrolysis to be ∼30 pN per FtsZ monomer, which is within the range of force required (8 – 80 pN) to drive cytokinesis as mentioned above (Lan et al., 2007; Hsin et al., 2012). Nonetheless, inward growth of the cell division septum likely contributes significantly to the constriction of the Z ring.

## Similarities and Differences Between Tubulin and FtsZ

Although FtsZ has minimal sequence similarity with tubulin, there are several regions that are highly similar in both proteins. Tubulin and FtsZ share only ∼10–18% sequence identity, yet both exhibit structural homology (de Pereda et al., 1996; Kusuma et al., 2019), suggesting convergent evolution (van den Ent et al., 2001; Battaje and Panda, 2017). Sequence alignment of FtsZ, α-tubulin and β-tubulin demonstrated that the T1 loop (common glycine), T4 loop (with the tubulin signature motif), T5 loop (common prolines), T6 loop (common asparagine), and T7 loop (common asparagine and aspartate) show high sequence identity. No significant sequence similarity was observed between tubulin and FtsZ after the T7 loop (Löwe, 1998; Löwe and Amos, 1998; Nogales et al., 1998a).

The secondary structure of both proteins contains a similar sequence of helices – strands – loops and follows similar nomenclature. FtsZ contains ten helices and ten strands. Although the secondary structures of both tubulin and FtsZ are quite similar, the two extra helices at the C-terminus and the C-terminal tail of tubulin are not long like those in FtsZ (Figure 1B) (Nogales et al., 1998a). In terms of tertiary structure, a structural prediction study by Pereda et al. involving 200 tubulin sequences and 12 FtsZ sequences from various organisms showed that FtsZ and tubulin have a nearly identical percentage of folds, helices and sheets (de Pereda et al., 1996). However, an *in silico* study that superimposed structures of different FtsZ proteins with tubulin showed that structural differences between FtsZ and tubulin are quite high, with an RMSD value of 8–10 Å (Kusuma et al., 2019). The NBDs of both proteins exhibit a Rossman fold topology (Löwe and Amos, 1998). Both FtsZ and tubulin belong to a distinct GTPase family, which bind to GTP and self-activate GTPase concomitant with polymerization (Nogales et al., 1998a). As suggested by the lack of sequence similarities, there is no structural homology in the C-terminal domains of both proteins. Superimposition of the structures confirmed that the IDC of FtsZ is also absent in tubulin (Battaje and Panda, 2017; Kusuma et al., 2019).

Despite sharing some structural similarities, FtsZ and tubulin diverge in how they form polymers. FtsZ protofilaments are formed by FtsZ monomers, whereas tubulin protofilaments consist of both α and β- tubulin monomers and require a gamma tubulin for nucleation and initiation of tubulin assembly. Another difference is that even if α and β- tubulins show similar degree of similarities with FtsZ, only the β- tubulin can exchange its GTP, whereas all FtsZ monomers can exchange their GTP and undergo GTP-dependent assembly. Furthermore, the C terminus of FtsZ ends with a β-sheet, whereas a helix is present at the C terminus of tubulin that is responsible for interaction with motor proteins (Figure 1B) (Erickson, 1998; Battaje and Panda, 2017). But the key structural difference between FtsZ and tubulin at the monomer level is the presence of the IDC only in FtsZ. This has been a boon for the discovery of compounds unique to FtsZ with minimal cytotoxicity toward eukaryotic cells.

## The Two Distinct Drug Binding Pockets of FtsZ

### The NBD

**FIGURE 2:**
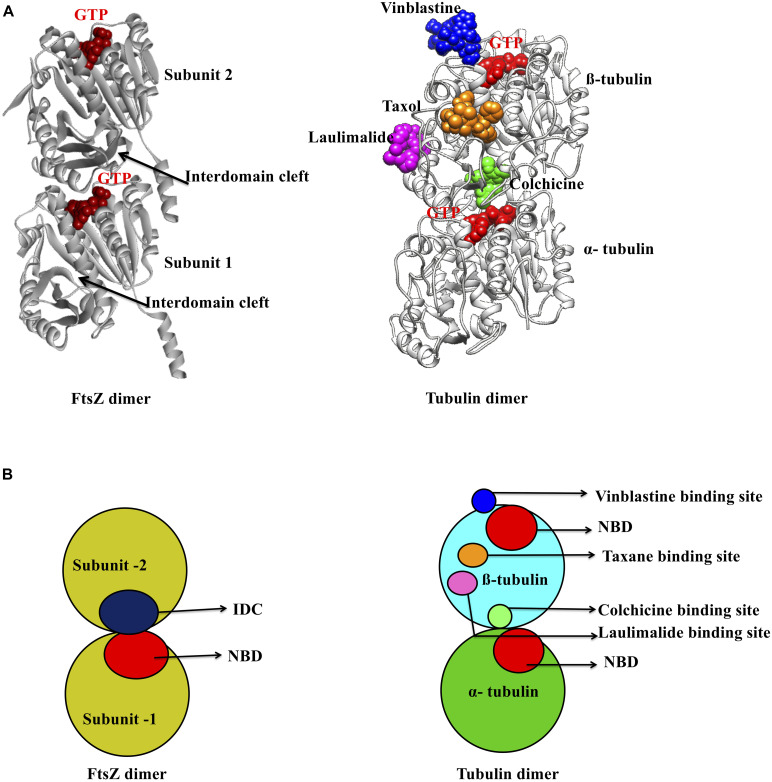
Major inhibitor binding sites in FtsZ and tubulin: **(A)** Structure of FtsZ and tubulin dimers with major drug binding sites. **(B)** Shown is a cartoon representation of the same.

As mentioned above, there are two main binding pockets for drug binding to FtsZ, the NBD and IDC (Figure 2). The NBD, at the interface between FtsZ monomers, includes helices H1–H6, sheets S1–S6 and the T1–T6 loop. It also includes the N-terminal part of H7 (Löwe, 1998; Panda et al., 2016). Seven distinct regions in FtsZ interact with GTP. The T1 loop interacts with the phosphate and the guanine base, whereas the T2 & T3 loops interact with the β- and γ-phosphates (Nogales et al., 1998a; Panda et al., 2016). The T4 loop contains the tubulin signature motif, which interacts with the α and β- phosphates (Mukherjee et al., 1993; de Pereda et al., 1996). The T5-loop interacts with the ribose sugar. The T6 loop possesses an asparagine residue (N165 in *E. coli*), which interacts with the guanine base via a hydrogen bond and is conserved in both FtsZ and tubulin (Panda et al., 2016). The guanine base is mainly recognized by amino acid residues present within the H7 helix. There are several small molecules that are known to interact with the FtsZ NBD (Table 1). Most of these could either interact with microtubules or were screened from a library of microtubule-interacting molecules. As the NBD is highly conserved in both FtsZ and tubulin, any molecules that bind to it may be toxic to mammalian cells. Indeed, curcumin, which binds the NBD of both FtsZ and tubulin, has an MIC of 100 μM in *B. subtilis* and an IC_50_ of 18 μM for HeLa cells (Rai et al., 2008; Chakraborti et al., 2011). Some other examples of this cross-toxicity are summarized in Table 1 (IC_50_/MIC).

**TABLE 1:** Reported FtsZ inhibitors, binding sites, and mechanisms of action.

Sl No. of core struct-ure^1^	Sl No. of drugs	Core structure	Drug	Binding site on FtsZ	Effects on assembly or GTPase activity^2^	IC_50_ in μ M (tubulin/microtubule/eukaryotic cells)/HC_50_	MIC in μ M	IC_50_ MIC ratio	References
A.	1.	Benzamide ring 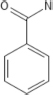	3-MBA and PC190723	IDC	E	> 180	2.81	> 64	Haydon et al., 2008
A.	2.		TXA707,TXA709 and TXA6101	IDC	E	> 233.25	3.9	> 60	Kaul et al., 2015; Fujita et al., 2017; Carro, 2019
A.	3.		3-substituted 2,6-difluorobenzamide derivatives **(I1)**	IDC	-	-	0.88–28.04	-	Bi et al., 2017
A.	4.		Isoxazole benzamide derivatives **(I2)**	IDC	E	> 331	0.04–10.4	> 32	Bi et al., 2018
A.	5.		1, 3, 4 -oxadiazol-2-one- benzamide derivatives **(I3)**	IDC	E	> 150	0.29–2.35	> 64	Bi et al., 2019
A.	6.		3-aminobenzamide derivatives **(I4)**	IDC	I	> 100	3.1	> 32	Lui et al., 2019
A.	7.		BOFP	IDC		-	1.32	-	Ferrer-Gonzalez et al., 2019
B.	8.	Quinoline and quinazoline ring 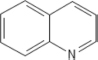	Berberine	NBD	I, G	18	95–380	0.047	Yu et al., 2005; Domadia et al., 2008; Raghav et al., 2017
A.	9.		Benzofuroquinolinium Derivatives **(I5)**	NBD	I, G	95.5	0.48–15.3	6.2	Zheng et al., 2018
A.	10.		9-phenoxy Berberine derivatives	IDC	I, G	-	4–65	-	Sun et al., 2014
A.	11.		Thiazole Orange Derivatives **(I6)**	IDC	E, G	96.5	1.36–5.45	17.7	Sun et al., 2017b
A.	12.		Indolyl-quinolinium derivatives **(I7)**	IDC	E	-	2.02–32	-	Cai et al., 2019; Carro, 2019
A.	13.		3-methylbenzo[d]thiazol-methylquinolinium **(I8)**	IDC	E, G	78.25	1.81–5.43	14.4	Sun et al., 2018
A.	14.		N-Methylbenzofuro[3,2-b] quinoline and Methylbenzoindole[3,2-b] quinoline derivatives, **(I9)**	IDC	I, G	-	4.16–12.5	-	Sun et al., 2017a
A.	15.		Thiazole-quinolinium derivatives **(I10)**	IDC	E	28.4	2.25	12.6	Li et al., 2015
C.	16.	Benzopyrone ring 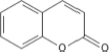	Coumarin and its derivatives	IDC	I, G	> 500	3420	> 0.14	Finn et al., 2001; de Souza et al., 2005; Duggirala et al., 2014
A.	17.		Polyketides compounds **(I11)**	IDC	G	-	5–40	-	Matsui et al., 2017
A.	18.		Quercetin dehydrate	IDC	I	> 100	378.5	> 0.26	Mathew et al., 2016
D.	19.	Phenylpropanoid 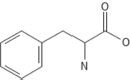	p-coumaric acid	IDC	I, G	215	122	1.7	Hemaiswarya et al., 2011; Lou et al., 2012; Chang and Shen, 2014
A.	20.		Cinnamic acid	IDC		2400	9000	0.27	Rastogi et al., 2008; Hemaiswarya et al., 2011; Niero and Machado-Santelli, 2013
A.	21.		Cinnamaldehyde	IDC		9.76	7560	0.0013	Domadia et al., 2007; Ng and Wu, 2011
A.	22.		Caffeic acid	IDC		> 100	1800	> 0.055	Rastogi et al., 2008; Hemaiswarya et al., 2011; Sanderson et al., 2013
A.	23.		Ferulic acid	IDC		500	> 515	< 1	Borges et al., 2013; Eroglu et al., 2015
E.	24.	Pyrimidine ring 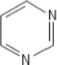	pyrimidine-quinuclidine derivatives	NBD	I	> 500	49.2	> 10	Chan et al., 2013; Haranahalli et al., 2016
A.	25.		2,4-disubstituted-6-thiophenyl-pyrimidine derivatives **(I12)**	NBD	I, G	> 128	4	>32	Fang et al., 2019
F.	26.	Complex ring structure	SB-RA-2001	IDC	E, G	45	5	9	Huang et al., 2006; Singh et al., 2014
		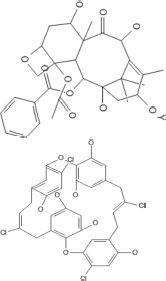							
	27.		Doxorubicin	IDC	I, G	8.3	40	0.20	Panda et al., 2015; Oncul and Ercan, 2017
	28.		Colchicine	IDC		6.5	160	0.04	Mathew et al., 2016
	29.		CCR11	IDC		18	3	6	Singh et al., 2012
	30.		Sulindac analog **(I13)**	IDC		> 100	19.5	> 5.1	Mathew et al., 2016
	31.		Bt-benzo-29	IDC		17	8	2.12	Ray et al., 2015
	32.		Tiplaxtinin	IDC	E	2.7	4.5	0.6	Elokdah et al., 2004; Sun et al., 2017c
	33.		Chrysophaentin A	NBD	I,G	> 150	4.6–9.26	> 32	Plaza et al., 2010; Keffer et al., 2013
	34.		UCM44	NBD	E	44	37.1	1.18	Ruiz-Avila et al., 2013
	35.		Biphenyl derivative **(I14)**	NBD	I	> 100	7	>14.28	Artola et al., 2015
G.	36.	Simple ring structure	Curcumin	NBD	I	18	100	0.18	Rai et al., 2008; Chakraborti et al., 2011
		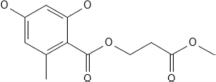							
	37.		Plumbagin	IDC	I,G	14.6	29	0.5	Acharya et al., 2008; Bhattacharya et al., 2013
	38.		*P. cataractum* SYPF 7131 bioactive compound **(I15)**	IDC	G	-	192.5–293	-	Wu et al., 2018
H.	39.	Peptide inhibitor	CRAMP	IDC	I	300	20	15	Ray et al., 2014
	40.		MciZ	Near IDC	I	-	-	-	Bisson-Filho et al., 2015

*^1^Non-peptide FtsZ inhibitors are classified into 7 groups based on their core ring structure (A–G). ^2^E, enhances assembly; I, inhibits assembly; G, inhibits GTPase activity; blank if unknown. **I1**: 3-((2-ethylhexyl)oxy)-2,6-difluorobenzamide, **I2**: 3-((5-(4-(Tert-butyl)phenyl)isoxazol-3-yl)methoxy)-2,6-difluorobenzami, **I3:** 2,6-Difluoro-3-((4-(4-bromophenyl)-5-oxo-1,3,4-oxadiazol-2- yl)methoxy)benzamide, **I4:** 2,6-Difluoro-3-(nonylamino)benzamide, **I5:** 5-Methyl-11-((3-(3-dipropylamine)-propylbenzo[d]thiazol-2(3H)-ylidene)methyl)benzofuro[3,2-b]quinolin-5-ium iodide, **I6:** 2-((E)-4- Hydroxystyryl)-1-methyl-4-((Z)-(3-methylbenzo[d]thiazol-2(3H)-ylidene)methyl)quinolin-1-ium iodide, **I7:** (E)-2-(2-(1H-indol-2-yl)vinyl)-1-methyl-4-(piperidin-1-yl)quinolin1-ium iodide, **I8:** 2-((E)-4-fluorostyryl)-1-methyl-4-((E)-(3-methylbenzo[d]thiazol-2(3H)-ylidene)methyl) quinolin-1-ium iodide, **I9:** 5-Methyl-11-(4-methoxyphenylamino)benzoindolo[3,2-b] quinolin-5-ium iodide**, I10:** Z)-1,2-Dimethyl-4-((3-(3-(4-methylpiperidin-1-yl)propyl)benzo[d]thiazol-2(3H)-ylidene)methyl)quinolin-1-ium iodide, **I11:** Gancaonin, **I12:** [1-(4-isopropylbenzyl)-4-(2-(2-(pyridin-4-yl)-6-(thiophen-2-yl)pyrimidin-4-yl)ethyl)-1,4-diazepane (Bb2)], **I13:** (Z)-N-(2-(dimethylamino)ethyl)-2-(5-fluoro-2-methyl-1-(4-(methylthio)benzylidene)-1H-inden-3-yl)acetamide, **I14:** [Biphenyl-3,5-diyl bis(3-hydroxybenzoate)], **I15:** Penicimenolidyu B.*

### The IDC

**FIGURE 3:**
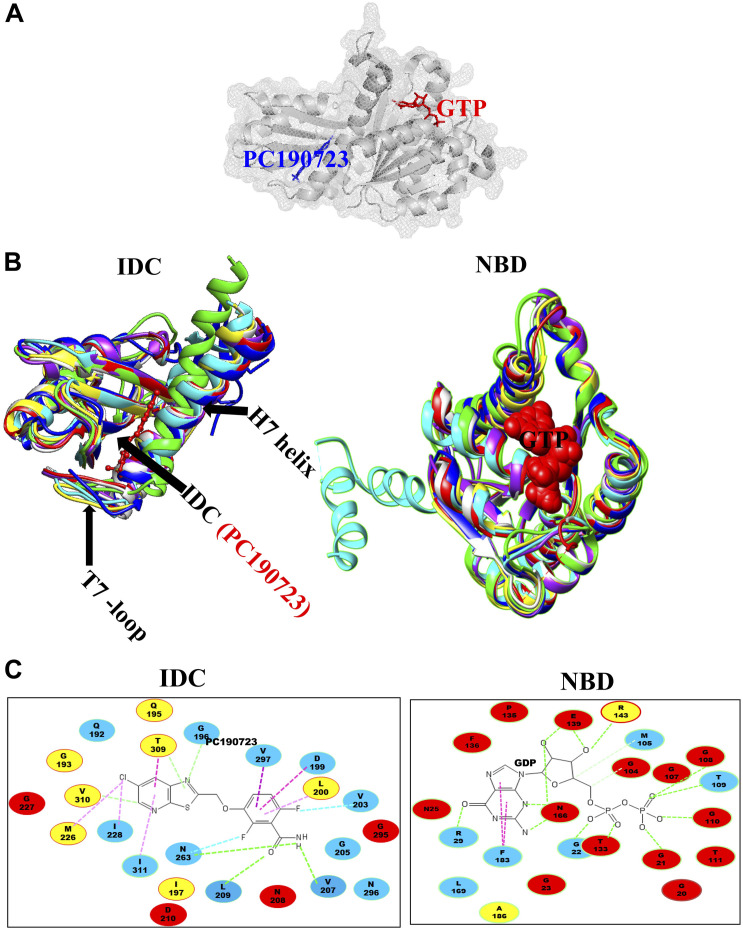
Structural alignment of GTP binding site and IDC in FtsZ. **(A)** IDC and NBD in FtsZ are denoted by mesh structures**. (B)** A structural level comparison of GTP binding sites and the IDC among FtsZs from *E. coli* (red), *B. subtilis (yellow), S. aureus (green), A. aeolicus (blue), P. aeruginosa (violet), M. jannaschii (cyan)*, and *M. tuberculosis (light gray)*. **(C)** Major residues around the GTP binding site and the IDC and their conservation in Gram positive species only (yellow), Gram negative species only (green) or in both Gram positive and Gram negative bacteria (red); non conserved residues are in blue.

The second major binding site in FtsZ that interacts with small molecules is the IDC, formed by the C-terminal half of the H7 helix, the T7 loop and the beta sheets in the C-terminal domain (Figure 1C) (Sun et al., 2014). The size of the cleft, the number of amino acid residues, their conservation and types vary among different bacterial species (Figure 3 and Table 2). For example, the IDC is less conserved in Gram negative bacteria (fewer than nine conserved residues) than Gram positive bacteria (more than nine conserved residues). The size of the IDC also varies among bacterial species, depending on the curvature of the H7 helix. For example, in the GTP bound state of *Staphylococcus aureus* FtsZ the curvature of the H7 helix decreases, which in turn increases the size of the cleft opening. The T7 loop of FtsZ also influences the cleft opening size. In bacteria such as *S. aureus* and *B. subtilis*, the T7 loop in the GTP bound state shifts downward, resulting in a larger cleft opening compared to the GDP bound state (Kusuma et al., 2019). As the T7 loop of one FtsZ subunit is inserted into the nucleotide-binding pocket of the adjacent FtsZ subunit to trigger GTPase activity (Panda et al., 2016), this loop is crucial for modulating FtsZ treadmilling dynamics.

**TABLE 2:** Roles of different IDC residues in binding ligands.

	IDC residues										
*Ec*FtsZ	G191	G195	E198	L199	M206	N207	V208	N263	S297	R307	T309
*Sa*FtsZ	Q192	G196	D199	L200	V207	N208	L209	N263	V297	V307	T309
*Bs*FtsZ	Q192	G196	D199	L200	I207	N208	L209	N263	V297	V307	T309
Thiazole ring	+	+	+	+	+	+	+	+	−	−	+
Quinoline ring	−	−	+	+	−	−	−	−	+	+	+
Benzopyrone	−	−	−	−	−	+	+	−	−	−	−
Phenylpropanoid	−	−	−	−	−	−	+	+	+	−	−
Naphthalene	+	+	+	−	−	−	−	+	−	+	+
Complex ring	+	−	+	+	−	−	+	−	+	−	−
Simple ring	−	−	−	+	−	−	+	+	−	−	+
Peptide	−	−	−	−	−	+	+	−	−	−	−

*(+) indicates bonding interaction; (−) indicates non-bonding residues (no interaction).*

In most bacterial species, many archaea, some chloroplasts and a few primitive mitochondria, the globular domains of FtsZ share 40–50% structural and functional similarity (Erickson, 1998; Tripathy and Sahu, 2019). Compared to the NBD, which exhibits 48–67% sequence identity among FtsZs from *E. coli, Pseudomonas aeruginosa*, *B. subtilis, S. aureus, Mycobacterium tuberculosis*, *Aquifex aeolicus*, and the archaeon *M. jannaschii*, the IDC shows less sequence conservation (34–59% identity) (Casiraghi et al., 2020). The three-dimensional structure of the NBD is quite similar among FtsZs from different bacterial species, whereas it is slightly different among various IDCs because of how the C-terminal beta sheets are organized (Kusuma et al., 2019).

The sequence level as well as structural level variability of IDCs among different FtsZs should facilitate the design of species-specific antibiotics (Casiraghi et al., 2020). For example, PC190723, an IDC inhibitor that will be described in greater detail below, is most effective against organisms that have a valine at the equivalent position of 307 in *E. coli* FtsZ, such as *S. aureus* and *B. subtili*s. In contrast, PC190723 is ineffective toward *E. coli* (Haydon et al., 2008), although inactivating the AcrAB efflux pump of *E. coli* significantly enhances susceptibility to the PC190723 prodrug TXY436, suggesting that resistance of Gram negative species to this compound is in part due its rapid clearance from the cytoplasm (Kaul et al., 2014).

Although the sequence and structure of the IDC as a whole is not highly conserved, components of the IDC, like the T7 loop, are highly conserved (de Pereda et al., 1996). Kusuma et al. compared the tertiary structure of staphylococcal and non-staphylococcal FtsZ proteins and showed that their structures differ mainly because of variations in the curvature of the H7-helix and organization of the C-terminal β-sheet (Kusuma et al., 2019). Superimposition of staphylococcal and non-staphylococcal FtsZs revealed that staphylococcal FtsZs were similar to each other, with an RMSD value of 0.3 Å, and non-staphylococcal FtsZs were more structurally variable among themselves (RMSD ∼1.3 Å). Conversely, *S. aureus* FtsZ (hereafter referred to as *Sa*FtsZ) showed much higher variation (RMSD ∼3 Å) when superimposed onto non-staphylococcal FtsZ. This difference was mainly due to the diversity in the arrangement of C-terminal β- sheets. Similarly, when compared the drug binding sites, both staphylococcal and non-staphylococcal FtsZ showed no significant structural differences in their GTP binding sites and T7 loops, whereas their IDCs were quite divergent. As the cleft opening size of the IDC and the curvature of helix H7 vary significantly between staphylococcal and non-staphylococcal FtsZs, compounds that bind to a staphylococcal FtsZ may not bind to non-staphylococcal FtsZ with similar affinity. Likewise, a compound targeting a non-staphylococcal FtsZ may not bind to other non-staphylococcal FtsZs with the same affinity (Kusuma et al., 2019).

### Comparing the IDCs of FtsZ With Tubulin

Microtubules, formed by polymerization of α-β- tubulin, are eukaryotic cytoskeletal proteins that play important roles in several cellular processes such as cell division, cell motility, intracellular transport and maintaining cell shape. Along with the GTP binding pocket, microtubules contain at least four major drug binding sites, including those for vinblastine, colchicine, laulimalide, and taxane (Figure 2A) (Lu et al., 2012). Vinblastine binds at the interface of the α-β- tubulin heterodimer (Figure 2B), which comprises the T7- loop, H10 and S9 strand of α tubulin and H6, the T5 loop and H6–H7 loop of β- tubulin (Steinmetz and Prota, 2018). The colchicine binding site includes the T7-loop, helices H7 and H8, and strands S8 and S9 of β- tubulin plus the T5 loop of α tubulin (Ravelli et al., 2004). The laulimalide binding site comprises helices H9 and H10 and the H10-S9 loop of β- tubulin, whereas taxol, one of three taxane derivatives commonly used as an anticancer drug, binds to the β- tubulin H7, S7, H6–H7 loop, S7–H9 (M-loop) and S9–S10 loop (Prota et al., 2014; Kellogg et al., 2017).

In contrast to this diversity of binding sites in microtubules, only the NBD and IDC of FtsZ have been identified as drug binding targets. The IDC of FtsZ consists mainly of the H7 helix, S7–S10 strands and T7 loop that structurally map to the taxol and colchicine binding sites in tubulin (Table 3 and Figure 2). Because the IDC has a lower level of sequence and structural similarity with tubulin compared with the NBD, colchicine and taxane can interact with FtsZ, but the interacting residues as well as binding affinity do not match with tubulin (White et al., 2002; Haydon et al., 2008). For example, although colchicine can bind to the IDC in FtsZ, the colchicine binding pocket of tubulin has no sequence similarity with the colchicine binding site in FtsZ from *M. tuberculosis* (White et al., 2002; Mathew et al., 2016). In another example, PC190723 (an IDC-specific inhibitor) binds to the taxol site on tubulin, but was >64-times more inhibitory to FtsZ than to tubulin (Haydon et al., 2008). Similarly, SB-RA-2001, a taxane derivative, binds to the IDC of *Bs*FtsZ, but when the binding site was superimposed onto the taxol site of tubulin, no identical residues were found; it also binds only very weakly to tubulin (Singh et al., 2014). Consequently, the drugs that bind to the IDC in FtsZ do not interact with tubulin with similar affinity and thus are less likely to be toxic to mammalian cells.

**TABLE 3:** Comparison of drug binding sites between FtsZ and tubulin.

Protein	Binding site	Secondary structures involved	References
FtsZ	NBD	H1–H7 helix, S1–S6 strands, and T1–T6 loops	Löwe, 1998
	IDC	H7 helix, S7–S10 strands, and T7 loop	Sun et al., 2014
Tubulin	NBD	H1–H7 helix, S1–S6 strands, and T1–T6 loops	Nogales et al., 1998a
	Colchicine	H7,H8 helix, S8,S9 strands, T7-loop : ß- tubulin T5-loop : α- tubulin	Ravelli et al., 2004; Steinmetz and Prota, 2018
	Taxane	H7 helix, S7 strand, H6–H7 loop, S7–H9 (M-loop) and S9–S10 loop : ß- tubulin	Kellogg et al., 2017
	Laulimalide	H9–H10 helix, H9–H9’ and H10–S9 loop : ß- tubulin	Prota et al., 2014
	Vinblastine	H6 helix, T5-loop and H6–H7 loop : ß- tubulin H10 helix, S9 strand, and T7-loop : α- tubulin	Steinmetz and Prota, 2018

### Molecular Insights Into the IDC

The N-terminal domains of FtsZ share high sequence identity in both Gram-positive bacteria (56–89%) and Gram-negative bacteria (43–84%), whereas lower sequence identities (30–70%) are shared within the C-terminal domains and IDCs. Inter-domain clefts of diverse FtsZs are composed of mostly hydrophobic residues along with a few polar and charged amino acids. Available crystallographic structures for protein-ligand interactions indicate that most small molecules prefer to bind to the hydrophobic pockets of their protein targets (Guo et al., 2015). Thus, the hydrophobic residues in the FtsZ IDC likely enhance the binding of organic molecules in aqueous environments, making the IDC a better target for small molecule inhibitors.

Some residues within the IDC are widely conserved in all bacterial species, some are conserved only among the Gram positive bacteria or in Gram negative bacteria, whereas other residues are specific to a particular species. For example, residues V189, Q192, G193, Q195, G196, I197, D199, L200, I201, V203, S204, G205, E206, V207, N208, L209, D210, M226, G227, I228, L261, M262, N263, T265, G295, T296, V297, T309, V310, and I311 are located within 6 Å of the IDC of *Sa*FtsZ. The corresponding residues in *Ec*FtsZ are shown in Table 4. Of these, N208, D210, G227, and G295 are conserved throughout bacteria that have FtsZ; G193, Q195, I197, L200, I201, M226, T309, and V310 are conserved mostly in Gram-positive species, and the remaining residues are not conserved (Figure 3C). These residues are involved in formation of different bonds with the small molecules–hydrogen bonds, hydrophobic, van der Waals, amide bonds or other types of interactions–and depend upon the chemical nature of the inhibitors and the interacting residues. For example, V207 and N263 are mainly involved in hydrogen bonding with PC190723, while L200 and I311 form hydrophobic interactions (Matsui et al., 2012). The size of the IDC, the number of amino acid residues and their types vary among different bacterial species. For example, a multiple sequence alignment of IDCs from 12 bacteria showed that 6–12 residues are conserved between Gram-positive and Gram-negative species, whereas more than 12 residues are conserved when aligned among only Gram positive bacteria.

**TABLE 4:** *Ec*FtsZ residues within the IDC and the corresponding *Bs*FtsZ and *Sa*FtsZ residues.

***Ec*FtsZ**	D187	V188	K190	G191	A192	Q194	G195	I196	E198	L199	R202	P203	G204	L205	M206
***Bs*FtsZ**	N188	V189	R191	Q192	G193	Q195	G196	I197	D199	L200	T203	P204	G205	L206	I207
***Sa*FtsZ**	N188	V189	R191	Q192	G193	Q195	G196	I197	D199	L200	V203	S204	G205	E206	V207



***Ec*FtsZ**	N207	V208	V213	M225	G226	S227	V229	L261	V262	N263	T265	A266	L270	R271	L272
***Bs*FtsZ**	N208	L209	V214	M226	G227	I228	I230	L261	M262	N263	T265	G266	L270	S271	L272
***Sa*FtsZ**	N208	L209	V214	M226	G227	I228	V230	L261	M262	N263	T265	G266	L270	S271	L272



***Ec*FtsZ**	F275	G295	T296	S297	L298	D299	P300	D301	M302	N303	E305	R307	T309	V310	V311
***Bs*FtsZ**	V275	G295	S296	V297	I298	N299	E300	N301	L302	K303	E305	V307	T309	V310	I311
***Sa*FtsZ**	A275	G295	T296	V297	I298	N299	P300	E301	L302	Q303	E305	V307	T309	V310	I311

**FIGURE 4:**
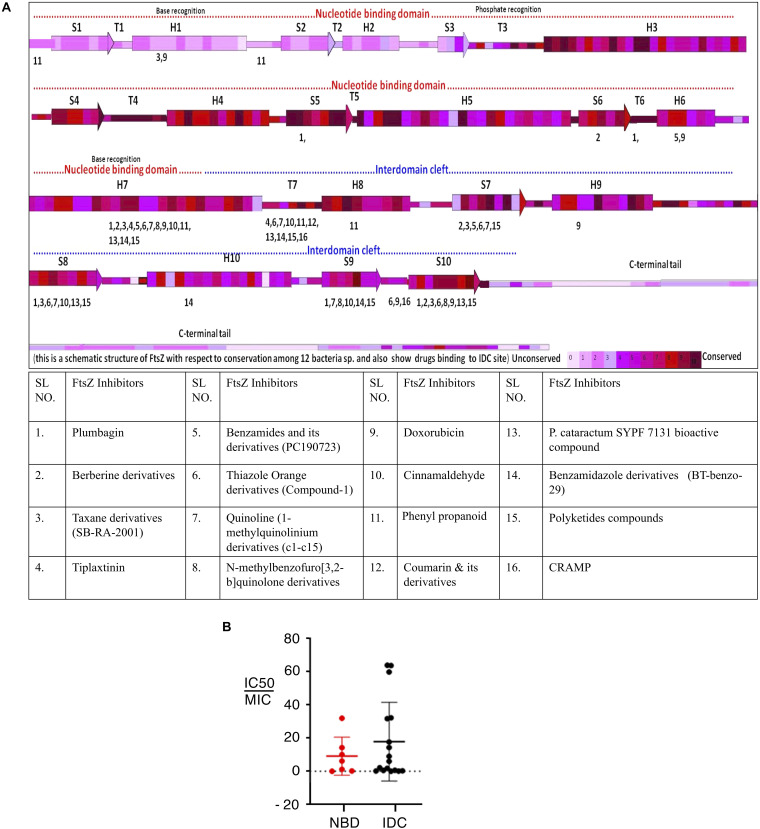
Interaction site for IDC inhibitors and their toxicity. **(A)** Schematic representation of FtsZ with respect to its conservation among 12 bacterial species and the interaction site of IDC inhibitors. **(B)** Comparison of IC_50_/MIC of both NBD and IDC inhibitors.

Although the IDC mostly consists of hydrophobic residues, it also contains several conserved hydrophilic residues that are important for interaction with small molecule inhibitors. Similarly, many residues are important for interaction with multiple inhibitors. For example, residues in *Ec*FtsZ such as G191, G195, L199, M206, N207, V208, N263, S297, R307 and T309 and their equivalent residues in both *B. subtilis* and *S. aureus* (Q192, G196, L200, V207, N208, L209, N263, V297, V307, and T309 in the latter species) mostly interact with more than one FtsZ inhibitor (Figure 4A and Table 2). Our analysis of the published literature suggests that most FtsZ inhibitors that target the IDC have higher IC_50_/MIC ratios than inhibitors that target the NBD (Figure 4B). Although IDCs from different bacteria are highly hydrophobic, their lower sequence conservation and variable cleft openings should potentially facilitate development of species-specific antibacterial agents.

### IDC Size and Conformational Flexibility

**FIGURE 5:**
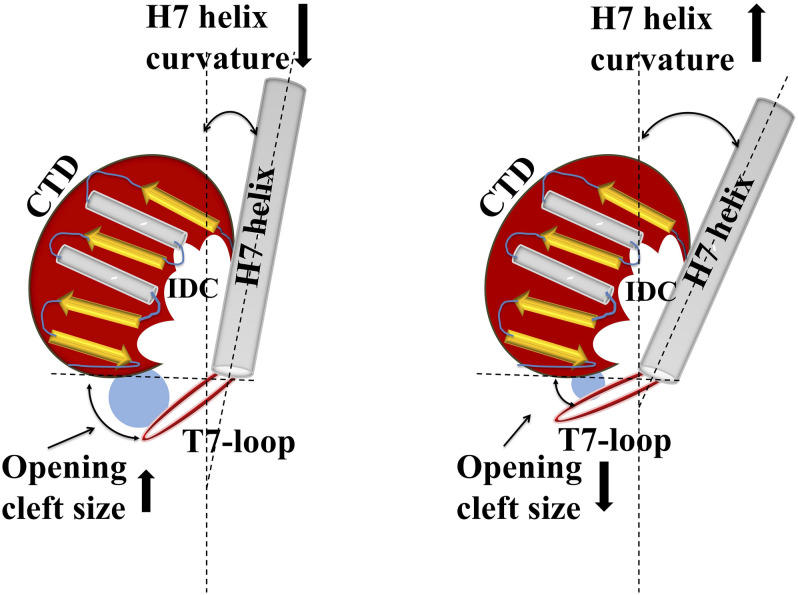
Structural relationship between IDC opening size and helix H7 of FtsZ. Cartoon representation shows how alterations in helix H7 curvature influence IDC opening size. Increased curvature of helix H7 yields a smaller cleft opening, whereas decreased H7 curvature leads to a larger cleft opening size.

Structural organization of the IDC indicates that the cleft has a specific size and a specific cleft opening. The cleft opening changes for different conformers of FtsZ such as GDP/GTP bound forms, and monomeric/polymeric FtsZ. Further, the size of the cleft opening differs in different bacterial species. Recent analysis of crystal structures of diverse FtsZs by Kusuma et al. indicated that IDC size depends upon the curvature of H7: if its curvature increases, the size of the IDC opening decreases, and vice-versa (Figure 5) (Kusuma et al., 2019). Using *in silico* analysis, they further measured the cleft opening size and the curvature of the H7 helix in *Staphylococcus* and non-*Staphylococcus* FtsZs, providing clues to the molecular accessibility of IDCs from different bacterial species (Kusuma et al., 2019). Their analysis shows that the curvature of H7 in *Sa*FtsZ is 140.3° (PDB ID: 3WGN) and the cleft opening size is 15.9 Å, whereas, in *Bs*FtsZ it is 164.5° (PDB ID: 2RHO) and 7.5 Å, respectively. The same study showed that the drug-binding pocket is also subject to species-level variations: *S. aureus* has the widest cleft opening (∼15 Å), whereas in *B. subtilis, M. tuberculosis, A. aeolicus, and P. aeruginosa* the cleft opening size is 9–10 Å. The IDC binder PC190723 has a size of 14.1 Å, with higher affinity for *Sa*FtsZ than *Bs*FtsZ. This wider cleft opening of *Sa*FtsZ vs. *Bs*FtsZ probably facilitates PC190723 entry and binding, and may be the major reason why it inhibits *S. aureus* cell division more effectively than that of *B. subtilis*.

Similarly, molecular dynamic simulations indicate that in the GTP bound state, the H7 helix is twisted backward and the T7 loop shifts downward, opening the cleft, whereas in GDP bound FtsZ the H7-helix is relaxed and the T7 loop shifts upward, closing the cleft’s opening. The size of the cleft opening in GDP and GTP bound *Sa*FtsZ varies between 15 and 20 Å (Kusuma et al., 2019). This model is supported by fluorescence anisotropy experiments showing that a fluorescent analog of PC190723, a nitrobenzoxadiazole probe, specifically binds to the polymeric form of FtsZ (Artola et al., 2017). Molecular dynamics simulations suggest that in monomeric FtsZ the cleft is in a closed or relaxed conformation, preventing the probe from interacting with FtsZ, whereas in polymeric FtsZ the cleft is in the open or tense conformation (Wagstaff et al., 2017; Schumacher et al., 2020), allowing interaction with FtsZ and resulting in fluorescence. *In silico* analysis of the PC190723 binding pocket in the IDC in different bacterial species showed that the microenvironment of the binding pocket affects the drug’s affinity toward FtsZ (Miguel et al., 2015). Analysis of FtsZ crystal structures and molecular dynamics trajectories showed that the conformation of the PC190723 binding pocket depends upon multiple factors such as bacterial species, genetic alterations, allosteric binding and polymerization state (Miguel et al., 2015). In particular, FtsZ polymerization and allosteric binding of the guanosine nucleotide may play a crucial role in stabilizing the PC190723 pocket. For example, for PC190723, the GDP-bound *Sa*FtsZ has a pocket score of -10.75 (PDB ID: 3VO8), whereas FtsZ without nucleotide has a pocket score of -4.29 (PDB ID 3VO9). Similarly, amino acid substitutions G193D, G196C, and N263K in *Sa*FtsZ change the microenvironment of the binding pocket, significantly affecting the binding of PC190723 and leading to drug resistance (Haydon et al., 2008; Miguel et al., 2015).

## IDC Inhibitors

Inhibitors that are known to interact with the IDC have been identified by molecular docking, simulation studies, mutational analysis, NMR and crystallographic studies. In addition, FtsZ inhibitors such as Ruthenium red, totarol, sanguinarine, OTBA, *Dichamanetin* and viriditoxin inhibit or promote FtsZ bundling, but the exact binding sites of these drugs on FtsZ are still not known (Santra et al., 2004; Beuria et al., 2005; Urgaonkar et al., 2005; Jaiswal et al., 2007; Beuria et al., 2009). Depending on their structure, we have characterized the IDC inhibitors in seven major groups, which are described below (Table 1).

## Benzamides

### 3-MBA, PC190723, and Derivatives

Ohashi et al. originally showed that a benzamide derivative, 3-MBA (3-Methoxybenzamide), inhibits the proliferation of bacteria by targeting FtsZ (Ohashi et al., 1999). Although 3-MBA has low antibacterial activity (MIC = 2048 μg/mL), it provided a strong starting point for FtsZ-targeted fragment-based drug discovery. Screening more than 500 benzamide analogs led to the discovery of the aforementioned PC190723, which contains a thiazolopyridine moiety fused to the benzamide by an ether linkage that makes it ∼2000 times more potent than the parent 3-MBA (MIC = 0.5–1 μg/ml). Molecular docking and X-ray crystallography demonstrated that PC190723 binds to the IDC, interacting specifically with R191, Q192, N263, V307, and T309 in *Bs*FtsZ (Haydon et al., 2008). Similarly, crystallography showed that PC190723 binding site in *Sa*FtsZ comprises Q192, G193,G196, I197, D199, L200, V203, G205, V207, N208, L209, M226, G227, I228, N263, G295, T296, T309, V310, and I311 (Table 4) (Matsui et al., 2012). PC190723 binding to the IDC disrupts the normal assembly of the Z ring by causing multiple FtsZ aggregates to distribute throughout the cell that are not able to form a coherent Z ring (Haydon et al., 2008).

Not surprisingly, *S. aureus* developed resistance to PC190723 by altering *Sa*FtsZ residues R191 G193, G196, V214, N263, or G266. G196 mainly interacts with the thiazolopyridine moiety and changes at this residue are commonly found in PC190723- resistant *S. aureus*. Interestingly, however, some PC190723-resistant mutants such as R191P and G196A in SaFtsZ and G196S in BsFtsZ remain sensitive to 3-MBA, suggesting that the less specific 3-MBA can still bind to an IDC pocket that occludes PC190723 binding (Adams et al., 2016). Moreover, some substitutions in FtsZ that render cells non-susceptible to PC190723, such as R191P, G193D, and G266S, at the same time confer benzamide dependence for normal cell division (Adams et al., 2016), although the mechanism for this drug dependent function of FtsZ is not clear. Interestingly, a 3-MBA-resistant mutant (A47) remains susceptible to PC190723 (Haydon et al., 2008).

A notable advance in optimizing benzamide action against FtsZ was the replacement of the chlorine atom on the pyridyl ring of PC190723 with a CF3 group, exemplified by a derivative called TXA707, which increases the drug’s metabolic stability and anti-staphylococcal activity (MIC: 0.25–2 μg/ml). Nonetheless, TXA707 is less effective on FtsZs with residue changes at G196 and others that mediate resistance, probably for the reason discussed above. A modification made in TXA707, in which a five membered oxazole and six membered phenyl ring (i.e., TXA6101) are flexibly linked, not only improved the binding affinity but also increased its activity against both wild-type methicillin resistant *S. aureus* (MRSA) and mutants carrying residue changes at FtsZ G196 (MIC = 0.125 μg/mL and 1 μg/mL, respectively) (Fujita et al., 2017). Crystallography and biochemical studies showed that both TXA707 and TXA6101 interact with the IDC. TXA6101 induces a conformational rearrangement of I197, M226, and I311 that leads to the formation of an inner hydrophobic pocket, with M226 acting as a gate that opens access to the pocket (Fujita et al., 2017).

Further advances have been made using improved benzamide prodrugs. TXY541 is a prodrug of PC190723 that is 143-times more soluble in an aqueous acid vehicle than PC190723 (Kaul et al., 2013). A prodrug of TXA707, TXA709, is structurally similar to TXY541 except that TXA709 contains a CF3 group instead of Cl group on the pyridyl ring, which increases the metabolic stability of the compound. Currently, TXA709 is in phase-I clinical trials. Recent reports showed that clinically isolated MRSA display resistance toward TXA709 at a frequency of 1 x 10^–8^, which is similar to that for PC190723. TXA709-resistant isolates carried mutations in FtsZ at G196S, N263K, G193D, G196C, and G196A, similar to residues that confer *S. aureus* resistance to PC190723 (Kaul et al., 2015).

**FIGURE 6:**
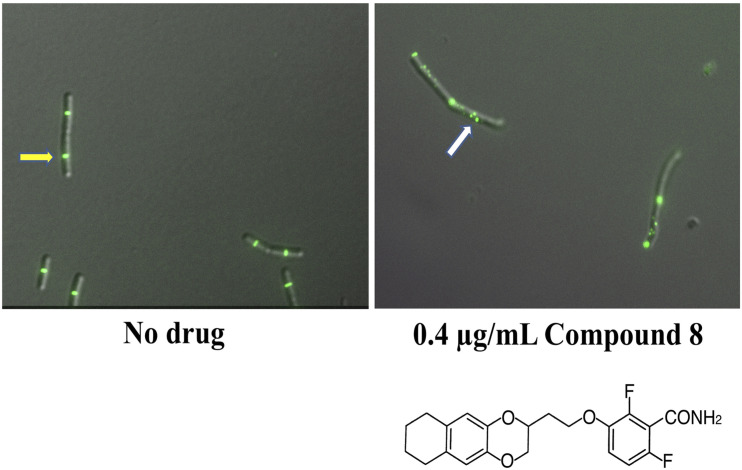
Benzamide derivatives disrupt Z-rings by redistributing FtsZ to multiple cellular foci. Shown are micrographs of *B. subtilis* cells with fluorescently labeled Z-rings, grown with either no drug added or after 90 min incubation with compound 8, a benzodioxane-benzamide derivative (diagrammed below). Reproduced from Straniero et al. (2021), under Creative Commons License CC BY 4.0. The yellow arrow highlights a functional and morphologically normal Z-ring; the white arrow highlights disrupted FtsZ localization as multiple foci.

In another study of benzamides, Bi et al. designed and synthesized a series of 3-substituted 2,6-difluorobenzamide derivatives, of which a chloroalkoxy derivative (**7)**, a 3-bromoalkoxy derivative (**12)** and a 3-alkyloxy derivative (**17)** exhibited good antibacterial activity against *B. subtilis* and susceptible/resistant *S. aureus* (Bi et al., 2017). Using structure-based drug design to target the IDC, they designed and synthesized a series of isoxazole (isoxazol-3-yl- and isoxazol-5-yl) containing benzamide derivatives. Some of these isoxazol-5-yl benzamide derivatives (B14) were ∼32 fold more potent against *B. subtilis* than PC190723 (Bi et al., 2018). In another study, Bi et al synthesized a series of 1, 3, 4-oxadiazol-2-one containing benzamide derivatives. Out of many derivatives, compound A14 showed the highest antibacterial activity against Gram positive bacteria (MIC 0.125-1 μg/mL) and less cytotoxicity against HeLa cells (IC_50_ > 64 μg/mL). *In silico* docking revealed that compound A14 binds to the IDC (Bi et al., 2019). A recent study of benzodioxane-benzamides identified a derivative (compound 8) with very high potency against MRSA and *B. subtilis*, with MICs at or below 0.1 μg/ml, good solubility, and very low toxicity to human cells. Like the original PC190723 (Haydon et al., 2008), compound 8 caused the delocalization of Z rings in *B. subtilis* into subcellular foci that were unable to function in cell division (Straniero et al., 2021) (Figure 6).

### 3-Aminobenzamide Derivatives

Using cell-based screening, Lui et al screened 47 derivatives of 3-aminobenzamide and showed that their compound 28 interacts with the FtsZ IDC. This compound exhibits high antibacterial activity (MIC 0.5–1 μg/mL against *S. aureus*) and less cytotoxicity (IC_50_ ≥ 100 μM, mouse L929 cell line) and worked in synergy with β-lactam antibiotics. Molecular docking studies showed that the C3 amino group of compound 28 interacts with the hydroxyl group of T309 in *Sa*FtsZ. The other *Sa*FtsZ residues in its proximity are G193, G196, M262, and N263 (Table 4). They also found that the M262I residue change is resistant to this molecule (Lui et al., 2019).

### Fluorescent Benzamide Derivatives That Bind to the IDC

Recently, Ferrer-González et al. developed a structure-guided fluorescent benzamide derivative by conjugating BODIPY to an oxazole benzamide FtsZ inhibitor (BOFP). BOFP binds to FtsZ from both Gram positive and Gram negative bacteria with K_*d*_s of 0.6–4.6 and 0.2–0.8 μM, respectively (Ferrer-Gonzalez et al., 2019). BOFP binds to the IDC, where the BODIPY moiety interacts with residues I228 and V307 of *Bs*FtsZ (Table 4). As it can label FtsZs within diverse bacteria, BOFP holds great promise for the screening of non-fluorescent FtsZ inhibitors and determining whether they perturb Z ring assembly in cells. In a recent study, Huecas et al. developed a competitive binding assay using specific high-affinity fluorescent probes to screen allosteric compounds that can interact with the IDC and inhibit FtsZ assembly. The probes displayed higher anisotropy in the presence of FtsZ polymer, where the IDC is open, compared with FtsZ monomers, where the IDC is closed. The specificity of this probe was assessed using a competitive assay with PC190723. The study demonstrated that the anisotropy of the probe decreased considerably upon binding of the IDC specific inhibitor PC190723, whereas it did not change upon binding of non-specific inhibitors. Thus, this probe can be used to identify inhibitors that specifically bind to the IDC (Artola et al., 2017; Huecas et al., 2021).

## Quinoline Ring compounds

Quinolinium derivatives have been widely used as therapeutics due to their antibacterial potency. Here, we describe recent developments in quinolinium molecules that target FtsZ. Berberine, a quinolinium derivative, was first described by Domadia et al. (2008) as an FtsZ interacting molecule and FtsZ inhibitor (Domadia et al., 2008). Since then, several other quinolinium derivatives were identified or synthesized that interact with the IDC and inhibit FtsZ function.

### Berberine and Its Derivatives

Berberine is a benzylisoquinoline alkaloid that has been used as an antimicrobial therapeutic for centuries. After identification of its interaction with FtsZ, Wong et al. used *in silico* structure-based design to synthesize a number of 9-phenoxyalkyl berberine derivatives that bind to the IDC of *Sa*FtsZ (Sun et al., 2014). A positively charged amine on these derivatives interacts with *Sa*FtsZ residue D199 and a C-9 methoxy binds to several hydrophobic residues (I228, V230 and V307) in the IDC. Modifications of these two moieties make the derivatives more potent than berberine (MIC = 100–400 μg/mL) and enable them to inhibit growth of MRSA and vancomycin-resistant Enterococci (VRE), with MICs of 2–8 μg/mL and 4–16 μg/mL, respectively. These compounds also exhibit moderate antimicrobial activity against Gram negative strains such as *E. coli* (MIC 32–128 μg/mL). Similarly, 9-phenoxy berberine derivatives inhibit FtsZ GTPase activity (IC_50_ 37.8 – 63.7 μM) more potently than the parent berberine molecule (IC_50_ = 272 μM) and showed similar effects on FtsZ polymerization. This confirms that the substitution of the 9-phenoxy group in berberine increases its affinity toward FtsZ and its antibacterial activity.

### Thiazole Orange Derivatives

Quinolines fused with a thiazole orange derivative confer broad spectrum antibacterial activities. Among them, 2-((E)-4- Hydroxystyryl)-1-methyl-4-((Z)-(3-methylbenzo[d]thiazol-2 (3H)-ylidene) methyl) quinolin-1-ium iodide (1) (compound-1) exhibits high antibacterial activity against *S. aureus* (MIC ∼ 1.5–3 μg/mL), other *staphylococci* (MIC ∼ 0.75–3.0 μg/mL) and *E. coli* (MIC ∼ 1.5 μg/mL). This compound enhances FtsZ bundling at lower concentrations (10 nm – 90 nm), inhibits GTPase activity (IC_50_ = 5 μg/mL) and is significantly less toxic to mammalian cells (IC_50_ = 98.15 μM). Molecular docking studies showed that it binds to the IDC of *Sa*FtsZ through both hydrophobic interactions at residues L200, M226, I228, L261, V297, L302, V307, and I311 and hydrogen bonding to V203 and L209 (Table 4) (Sun et al., 2017b).

### Quinolinium and Quinolone Derivatives

High throughput phenotypic screening by the NIH screened 215,110 molecules against the *M. tuberculosis* (Mtb) H37Rv strain and the data are freely available. Using the results from this screen, Mathew and coworkers found that quinoline and quinazoline can inhibit *Mtb*FtsZ functions (Mathew et al., 2013). Subsequently, Cai et al. synthesized a series of 1-methylquinolinium derivatives (c1-c15) by combining an indole fragment at the 2-position with different amino groups at the 4-position (Cai et al., 2019). These compounds strongly inhibited FtsZ activity and growth of MRSA and VRE, with MIC values between 1 and 4 μg/mL. C2 and c9 derivatives enhanced bactericidal activity with an MIC of 1 μg/mL in *S. aureus* (ATCC 29213). Both compounds possess a common piperidine group at the 4-position of the 1-methylquinolinium core that might increase its antibacterial properties compared with other indole-quinolinium derivatives. Molecular docking studies predicted that these derivatives bind to the IDC of *Sa*FtsZ mostly through hydrophobic interactions with residues Q192, G196, L200, V203, L209, M226, G227, I228, and V297 and electrostatic interaction with D199 (Table 4).

Several quinolone and quinoline derivatives exhibited antibacterial activity against Gram positive and Gram negative bacteria (Piddock and Walters, 1992; Aldred et al., 2014; Zhang et al., 2018). Sun et al. synthesized a series of quinoline derivatives containing the unique quaternary pyridinium core, many of which demonstrated antibacterial activities (Sun et al., 2017a). These compounds interacted hydrophobically with FtsZ residues D199, L200, V297, and V307, and the imino group of these derivatives could hydrogen-bond with FtsZ T309. These compounds showed ∼50-fold better antibacterial activity against *B. subtilis* (MIC ∼2–8 μg/mL) compared with berberine (MIC = 128 μg/mL). In their next study, Sun et al. synthesized sixteen 3-methylbenzo[d]thaizol-methylquinolinium derivatives with different groups added to the ortho-position of the 1-methylquinolinium core (Sun et al., 2018). One of the derivatives, A2, showed strong antibacterial activity (MIC = 1.5 μg/mL) by inhibiting FtsZ functions, and like the others, exhibited low toxicity toward mammalian cells (IC50 = 78.25 μM) (Sun et al., 2018). A docking study showed that A2 interacts with residues D199, L200, M226, I228, V297, T309, and I311 of *Sa*FtsZ (Sun et al., 2018).

From previous studies, it was clear that thiazole and quinolinium groups are important for antibacterial activity. Li et al. synthesized various thiazole-quinolinium derivatives and evaluated their antibacterial activities against Gram positive and Gram negative species (Li et al., 2015). All compounds showed good antibacterial activity (MIC 1–32 μg/mL) against *S. aureus.* A methyl group substitution at the quinolinium ring resulted in better antibacterial potency than the bulky indolyl group. These derivatives were effective against antibiotic resistant strains, did not induce antibiotic resistance, and showed less cytotoxicity toward mammalian cells (16HBE, HK-2 L929 with IC50 12–26 μg/mL). Molecular docking studies determined that thiazole-quinolinium derivatives interact with the IDC through numerous hydrophobic bonds with residues D199, L200, M226, I228, V297 and van der Waals interactions with Q195, V310, G205, and I311 of *SaFtsZ* (Table 4).

## Benzopyrone Ring Compounds (Coumarin and Its Derivatives)

Compounds harboring a benzopyrone ring are known to inhibit assembly and GTPase activity of FtsZ. Coumarin (1, 2- benzopyrone) is a natural polyphenolic compound with a benzopyrone ring (Detsi et al., 2017). Duggirala et al. screened several natural compounds including benzopyrone derivatives and showed that coumarins, specifically scopoletin and daphnetin, inhibit FtsZ polymerization and GTPase activity. Molecular docking studies showed that coumarin binds to the IDC via its interaction with highly conserved amino acids such as N207, D209, and D212 in the T7 loop of *Ec*FtsZ (Duggirala et al., 2014). In the case of scopoletin, the hydroxyl group of coumarin interacts with *Ec*FtsZ residue G204 and the keto group interacts with N207 via hydrogen bonding, whereas daphnetin interacts with G104. In other coumarin derivatives such as umbelliferone and 7-diethylamino-4-methyl coumarin, an oxygen group interacts with N207 and F210 of *Ec*FtsZ. The anti-tubercular activity of coumarin derivatives is reviewed elsewhere (Keri et al., 2015). Apart from the IDC, coumarin derivatives might also interact with the NBD of FtsZ in different organisms. Molecular docking studies revealed that most coumarin derivatives interact with the NBD of *Mycobacterium smegmatis* FtsZ via hydrogen bonding with residues N41, G103 and R140 (Sridevi et al., 2017).

## Phenylpropanoids (Cinnamaldehyde and Its Derivatives)

Plant-derived natural products are attractive for antibiotic development because they often are less toxic to mammalian cells. Phenylpropanoids are a group of natural organic compounds that are synthesized by plants using phenylalanine and tyrosine. Most phenylpropanoid derivatives possess antibacterial activity, and include cinnamic acid, p-coumaric acid, caffeic acid, chlorogenic acid, eugenol, and ferulic acid (Puupponen-Pimia et al., 2001; Hemaiswarya and Doble, 2009, 2010). These compounds inhibit GTPase activity of FtsZ and are able to disassemble preformed FtsZ polymers with varying effectiveness. For example, the IC_50_ values of FtsZ assembly for eugenol, ferulic acids and 3, 4-dimethoxycinnamic acids are more than 250 μM, whereas cholinergic acid, cinnamic acid, p-coumaric acid and caffeic acid have IC_50_ values of 70 μM, 238 μM, 190 μM, and 106 μM, respectively.

Molecular docking studies indicate that all the phenylpropanoids interact with the T7-loop of IDC. For example, chlorogenic acid, 3, 4-dimethoxycinnamic acid, 2, 4, 5-trimethoxycinnamic acid and ferulic acid interact with residues A11, G36, N207, V208, D209, and F210 of *Ec*FtsZ via hydrogen bonds and P203, N207 *via* hydrophobic bonds (Hemaiswarya et al., 2011). Other phenylpropanoids such as cinnamic acid, p-coumaric acid and caffeic acid bind to M206 and T296 of *Ec*FtsZ through hydrogen bonding. Among phenylpropanoid derivatives, chlorogenic and caffeic acid possesses two hydroxyl groups on their benzene ring, making them more hydrophilic than the other compounds containing methoxy substituents, resulting in higher affinity toward FtsZ (Hemaiswarya et al., 2011). Thus, the presence of hydroxyl groups in phenylpropanoids favor hydrogen bonding with the side chains of FtsZ active site residues that makes the compounds more effective.

Cinnamaldehyde, a phenylpropanoid, exhibits broad spectrum antibacterial activity against diverse species such as *E. coli* (MIC ∼ 1000 μg/mL), *B. subtilis* (MIC ∼ 500 μg/mL) and MRSA (MIC ∼ 250 μg/mL) (Domadia et al., 2007). It contains an aromatic benzene ring with an α, β- unsaturated carbonyl moiety and inhibits FtsZ assembly and GTPase activity in a dose dependent manner (Li and Ma, 2015). *In silico* docking and STD NMR spectroscopy showed that H2 and H3 of cinamaldehyde interact with residues G295 and V208 of FtsZ. The aromatic ring of cinamaldehyde is in close proximity to the aliphatic side chains of residues F203, M206, N207, and V208, whereas its carbonyl group is in close proximity to the side chain of N203, the guanidium group of R202 and the hydroxyl group of S297 (Domadia et al., 2007). These studies suggest that cinnamaldehyde preferably interacts with the IDC of FtsZ. Furthermore, multiple sequence alignment shows that the cinnamaldehyde-interacting residues such as G295, V208, R202, N263, and S297 are conserved among FtsZs from different bacterial species.

## Taxane Ring Compounds

The structural kinship between FtsZ and tubulin suggest that some microtubule targeting drugs might also target FtsZ, and taxanes are attractive candidates. Indeed, a screen of 120 taxane derivatives identified several taxanes that bind to FtsZ and exhibit effective anti-tubercular activity (Huang et al., 2006). Among those, SB-RA-2001, a derivative of a non-cytotoxic taxane, contains a 3-naphtha-2yl acryloyl group at the C13 position and showed promising anti-tubercular activity against both drug sensitive and resistant *M. tuberculosis* strains. *In silico* docking studies revealed that this compound binds to the IDC of FtsZ at the PC190723 interaction site (Singh et al., 2014). The SB-RA-2001 binding pocket in *Bs*FtsZ includes residues R29, E32, N33, N188, R191, Q192, Q195, G196, D199, I230, N263, T265, N299, N301, L302, K303, E305, V307, T309 (Table 4) of which many are present in the IDC. Its major interaction with *Bs*FtsZ is via hydrogen bonding with residues E305, R191, Q192, N188, and N33. Structural alignment of the taxane binding site on FtsZ and the paclitaxel binding site on tubulin indicated that no identical residues exist between these two sites.

## Other Small Molecules With Simple and Complex Ring Groups

Apart from molecules discussed above, there are several other small molecules with different size and ring structures that are reported to interact with the IDC.

### Plumbagin

Plumbagin (5-hydroxy-2-methyl-4, 4-naphthoquinone) is a naturally occurring naphthoquinone originally isolated from the plumbago plant (de Paiva et al., 2003; Aziz et al., 2008). It inhibits proliferation of diverse species such as *S. aureus, P. aeruginosa*, *B. subtilis* (MIC∼29 μM), *Proteus vulgaris* and *M. smegmatis* (MIC ∼ 31 μM) (de Paiva et al., 2003; Mathew et al., 2010). Plumbagin binds to *Bs*FtsZ and inhibits its assembly and GTPase activity (Bhattacharya et al., 2013). *In vitro* and *in silico* assessment studies demonstrated that the plumbagin binding site is distinct from the NBD in *Bs*FtsZ (Bhattacharya et al., 2013). The residues of *Bs*FtsZ that constitute the plumbagin binding site include the H7 helix and other residues in the IDC such as R191, Q192, Q195, G196, D199, N263, T265, N299, V307, and T309 (Table 4). Of these, R191, Q195, D199, and N299 of *Bs*FtsZ are involved in hydrogen bonding with plumbagin (Bhattacharya et al., 2013). Mutational studies confirmed that D199 and V307 of *Bs*FtsZ play an important role in plumbagin binding. *In silico* studies showed that the plumbagin binding site in *Ec*FtsZ includes residues G21, M104, T132, P134, E138, R142, N165, F182, A185, and L189 (Bhattacharya et al., 2013). Notably, these residues are in a completely different part of FtsZ than the plumbagin binding pocket of *Bs*FtsZ, suggesting that FtsZs of different bacteria may have different ligand binding properties.

### Fungal Compounds

Since the discovery of penicillin, it is well known that bioactive molecules of fungi show strong antimicrobial properties. While screening 58 fungal compounds from 24 different genera, Wu et al. found that *Penicillium cataractum* SYPF 7131 has strong antibacterial activity against *S. aureus* (Wu et al., 2018). Out of the 8 known and unknown isolates from its fermentation broth, 3 compounds showed effective antibacterial activity (MIC 10–65 μg/mL) and strong interaction with FtsZ. An *in silico* study suggested that these compounds interact with the IDC of *Sa*FtsZ by hydrogen bonding with residues G205, N263, T309, L209, G196, G227, and G193, and hydrophobic interaction with L200, L209, I311 L261, V307, V203, I228, I311 V297, and V203 (Table 4).

### Doxorubicin

Doxorubicin is an anthracycline antibiotic which inhibits bacterial proliferation with moderate inhibition against *E. coli* (MIC 40 *μ*M) and strong inhibition against *S. aureus* (MBC 5 *μ*M). In the presence of doxorubicin, *E. coli* becomes highly filamentous without affecting chromosome segregation, indicating a cell division defect. Panda et al. showed that doxorubicin binds to a site in FtsZ distinct from the NBD. The amino sugar region of doxorubicin sits in a polar cavity and involves hydrogen-bond interactions with E32, R33, and D187 of *Ec*FtsZ, whereas the ethyloxy side chain involves hydrogen-bond interaction with E305 (Panda et al., 2015). The hydrophobic part of the molecule (the aromatic rings) sits in a cavity lined by hydrophobic residues of FtsZ, *e.g.*, V171, V188, M225, V229, and L248. The binding site contains many highly conserved residues, including E32, R33, V171, D187, V188, M225, G226, P247, L248, M302, N303, E305, and R307.

### CCR-11

Rhodanine derivatives can perturb the assembly of FtsZ polymers and inhibit bacterial proliferation. Singh et al. screened a library of 151 rhodanine derivatives, of which 8 compounds showed good antibacterial activity (MIC ∼2 μM) and 3 specifically inhibited division of *B. subtilis* cells (Singh et al., 2012). One of these molecules, CCR-11, interacts with FtsZ with a Kd of 1.5 ± 0.3 μM and inhibits FtsZ assembly and GTPase activity. Docking studies revealed that CCR-11 binds to the IDC. The fluorine atoms of the CCR-11 trifluoromethylphenyl side chain interact with *Bs*FtsZ T203 and CCR-11 also interacts with *Bs*FtsZ G205, I207, L272, V275, and I298 through hydrophobic interactions. The thiazolidine ring of CCR-11 interacts with T203 and D199 of *Bs*FtsZ. CCR-11 inhibited HeLa cell proliferation with an IC_50_ value of 18.1 ± 0.2 μM, which is 6 times higher than the MIC (3 μM) of CCR11 on *B. subtilis* (Singh et al., 2012).

### Bt-Benzo-29

Ray et al. screened 100 benzamidazole compounds for them) ability to elongate *B. subtilis* cells, out of which one compound, N-(4-sec-butylphenyl)-2- (thiophen-2-yl)-1H-benzo[d]imidazole-4-carboxamide (BT-benzo-29), causes cell filamentation. BT-benzo-29 inhibits FtsZ functions by interacting with FtsZ (Kd = 24 ± 3 μM) and inhibits proliferation of *B. subtilis* with an MIC of 17 μM (Ray et al., 2015). A molecular docking study proposed that BT-benzo-29 binds to the *Bs*FtsZ C-terminal portion of the globular domain, near the T7 loop. The interaction involves hydrogen bonding with L206 and S296 and hydrophobic interactions with D199, T203, P204, G205, L206, N208, L270, S271, L272, V275, S296, V297, I298, and E300 residues (Ray et al., 2015). Mutational studies showed that L272A and V275A mutants had weaker inhibitory effects on the assembly and GTPase activity. Unfortunately, BT-benzo-29 inhibits HeLa cell proliferation with an IC_50_ 17 ± 2 μM, only ∼4 times higher than the IC_50_ for *B. subtilis*.

### Tiplaxtinin

Tiplaxtinin is an indole oxoacetic acid derivative (Elokdah et al., 2004). Using a cell based screen of 250 compounds, Sun et al. identified Tiplaxtinin as a bacterial cell division inhibitor (Sun et al., 2017c). Tiplaxtinin has strong antibacterial activity against Gram-positive pathogens, with MICs of 4.55–9.10 μM (2–4 μg/mL). Both *in vitro* and *in vivo* findings indicate that tiplaxtinin is capable of effectively disrupting dynamic assembly of FtsZ, GTPase function and Z-ring formation; tiplaxtinin-induced multiple FtsZ foci in *B. subtilis* cells is similar to the *in vivo* effects of benzamides. Molecular docking studies of this compound in *Sa*FtsZ revealed that tiplaxtinin binds near the T7-loop and H7 helix in the IDC region. The trifluoromethoxy group of tiplaxtinin forms hydrogen bonds with G193 and G227 and halogen bonds with V189, Q192, G193, and M226 (Sun et al., 2017c). Similarly, the carbonyl group interacts with T265 through a hydrogen bond. Tiplaxtinin also interacts with V189, Q192, G193 D199, L200, L209, M226, G227, I228, and V297 residues via hydrophobic interactions (Table 4).

## Small Inhibitory Peptides That Bind Near the IDC

Cathelin related antimicrobial peptide (CRAMP) is present in multicellular organisms and helps the innate immune system in the fight against microbes (Bergman et al., 2006). Like many antimicrobial peptides, CRAMP has an amphipathic α helical conformation. The active part of CRAMP consists of 18 amino acid residues from 16 to 33 (GEKLKKIGQKIKNFFQKL), which inhibits bacterial proliferation (MIC 20–50 μM) and GTPase activity of FtsZ in concentration dependent manner (IC50 ∼ 70 ± 14 μM) (Ray et al., 2014). Molecular docking studies suggest that both hydrophobic and hydrophilic amino acid residues of CRAMP can bind to the T7 loop (L206, I207, N208, and D210) and C-terminal residues adjacent to T7 loop. CRAMP binding to FtsZ is stabilized through salt bridges, hydrogen bonding, hydrophilic and hydrophobic interactions. K25 of CRAMP binds to D210 of T7 loop through a salt bridge and the G16, K27, K32 of CRAMP form hydrogen bonds with both R286 and D287 residues of FtsZ.

Another small FtsZ-inhibitory peptide is MciZ, a 40-aa peptide produced during sporulation of *B. subtilis*. MciZ interacts directly with FtsZ, inhibiting FtsZ polymerization and Z ring assembly *in vivo* (Handler et al., 2008; Ray et al., 2013). Using crystallography and computational techniques, Bisson-Filho et al. demonstrated that MciZ interacts with the C-terminal domain of FtsZ and thus does not bind to the NBD (Bisson-Filho et al., 2015). However, MciZ does not bind to the IDC either, and instead interacts with H10 and beta strand 9 of FtsZ. This results in occlusion of subunit-subunit contacts that causes capping of the growing FtsZ protofilament end (Araujo-Bazan et al., 2019). Other peptide inhibitors of FtsZ, including Kil from bacteriophage lambda and GP0.4 from bacteriophage T7, disrupt assembly of *Ec*FtsZ protofilaments, but their binding sites on FtsZ are not yet known (Kiro et al., 2013; Haeusser et al., 2014; Hernandez-Rocamora et al., 2015).

## Conclusion and Future Perspectives

We have described many small molecules that can interact with the IDC of FtsZ. Despite structurally mapping to the taxol and colchicine binding sites in tubulin, the IDC shares a low level of sequence and structural similarities with tubulin, reducing the likelihood that small molecules targeting the IDC will be toxic to mammalian cells. Molecules targeting the FtsZ NBD, on the other hand, are likely to have adverse effects on tubulin, and thus mammalian cells.

In most species, the IDC in FtsZ extends from residues 186 to 320 in *Ec*FtsZ. However, there are only a handful of residues that are specifically involved in interacting with small molecules (Table 2). Many of these residues are hydrophobic and favor interaction with small molecules, making the IDC a good druggable site. Fortunately, a few of these residues are conserved in both Gram positive and Gram negative bacteria and are essential for FtsZ functions. For example, G191 of *Ec*FtsZ is important for FtsZ assembly and G193 and G196 of *Sa*FtsZ are essential for interaction with anti-FtsZ drugs. Not surprisingly, alterations to any of these residues either inhibit FtsZ assembly or result in drug resistance. The G196A substitution in *Sa*FtsZ remains sensitive to 3-MBA despite conferring resistance to PC190723, suggesting that acquiring drug resistance comes with a fitness cost. It is notable that the V307 residue in FtsZ is important for FtsZ interaction with several drug classes including benzamides, plumbagin, quinolines, taxanes (SB-RA-2001), and berberine derivatives. Likewise, the T7-loop residue M206 of *Ec*FtsZ interacts with phenylpropanoid derivatives, and additional residues in the T7-loop interact with other drug molecules. Although the specter of resistant mutations is a significant challenge, molecules targeting the T7 loop may be less likely to induce resistant mutations because of its requirement in GTP hydrolysis.

Apart from the differences in residues among bacterial FtsZs, the size of the IDC and the cleft opening also varies in different bacterial species, with PC190723 binding to the larger cleft opening in *Sa*FtsZ with high affinity, and to the smaller cleft opening in *Bs*FtsZ with very low affinity. As a result, compounds can be tailored for specific species by targeting their IDCs. The limited number of FtsZ crystal structures and the lack of understanding of drug binding pockets in FtsZ have so far hindered such fine tuning, and consequently anti-FtsZ drugs are not yet ready for the clinic. Most of the IDC-drug interaction studies rely on *in silico* studies, whereas only a few drug molecules such as PC190723 and some of its derivatives have been subject to experimental genetic and structural studies. Nonetheless, recently reported derivatives of PC190723 exhibit very low MICs on important Gram-positive pathogens and have low toxicity profiles. Targeting FtsZs in Gram-negative pathogens will be more challenging because of increased barriers to permeability due to the outer membrane, but the small size of many of the compounds reviewed here, along with combination therapy using adjuvants that perturb the outer membrane and/or drug efflux pumps, provide promising future avenues (Khare et al., 2019). Continued development of better small molecule inhibitors that target FtsZ, as well as discovery of small molecules that can inhibit the activity of other conserved bacterial cell division proteins, will require continued collaboration between medicinal chemists, structural biologists and microbiologists.

## Author Contributions

All authors listed have made a substantial, direct and intellectual contribution to the work, and approved it for publication.

## Conflict of Interest

The authors declare that the research was conducted in the absence of any commercial or financial relationships that could be construed as a potential conflict of interest.

## Publisher’s Note

All claims expressed in this article are solely those of the authors and do not necessarily represent those of their affiliated organizations, or those of the publisher, the editors and the reviewers. Any product that may be evaluated in this article, or claim that may be made by its manufacturer, is not guaranteed or endorsed by the publisher.

## References

[B1] Acharya B. R. Bhattacharyya B. Chakrabarti G. (2008). The natural naphthoquinone plumbagin exhibits antiproliferative activity and disrupts the microtubule network through tubulin binding. *Biochemistry* 47 7838–7845. 10.1021/bi800730q 18597479

[B2] Adams D. W. Wu L. J. Errington J. (2016). A benzamide-dependent ftsZ mutant reveals residues crucial for Z-ring assembly. *Mol. Microbiol.* 99 1028–1042. 10.1111/mmi.13286 26601800 PMC4832351

[B3] Addinall S. G. Bi E. Lutkenhaus J. (1996). FtsZ ring formation in fts mutants. *J. Bacteriol.* 178 3877–3884. 10.1128/jb.178.13.3877-3884.1996 8682793 PMC232649

[B4] Aldred K. J. Kerns R. J. Osheroff N. (2014). Mechanism of quinolone action and resistance. *Biochemistry* 53 1565–1574. 10.1021/bi5000564 24576155 PMC3985860

[B5] Allard J. F. Cytrynbaum E. N. (2009). Force generation by a dynamic Z-ring in *Escherichia coli* cell division. *Proc. Natl. Acad. Sci. U.S.A.* 106 145–150. 10.1073/pnas.0808657106 19114664 PMC2629190

[B6] Anderson D. E. Gueiros-Filho F. J. Erickson H. P. (2004). Assembly dynamics of FtsZ rings in *Bacillus subtilis* and *Escherichia coli* and effects of FtsZ-regulating proteins. *J. Bacteriol.* 186 5775–5781. 10.1128/JB.186.17.5775-5781.2004 15317782 PMC516820

[B7] Araujo-Bazan L. Huecas S. Valle J. Andreu D. Andreu J. M. (2019). Synthetic developmental regulator MciZ targets FtsZ across *Bacillus* species and inhibits bacterial division. *Mol. Microbiol.* 111 965–980. 10.1111/mmi.14198 30636070

[B8] Artola M. Ruiz-Avila L. B. Ramirez-Aportela E. Martinez R. F. Araujo-Bazan L. Vazquez-Villa H. (2017). The structural assembly switch of cell division protein FtsZ probed with fluorescent allosteric inhibitors. *Chem. Sci.* 8 1525–1534. 10.1039/c6sc03792e 28616148 PMC5460597

[B9] Artola M. Ruiz-Avila L. B. Vergonos A. Huecas S. Araujo-Bazan L. Martin-Fontecha M. (2015). Effective GTP-replacing FtsZ inhibitors and antibacterial mechanism of action. *ACS Chem. Biol.* 10 834–843. 10.1021/cb500974d 25486266

[B10] Aziz M. H. Dreckschmidt N. E. Verma A. K. (2008). Plumbagin, a medicinal plant-derived naphthoquinone, is a novel inhibitor of the growth and invasion of hormone-refractory prostate cancer. *Cancer Res.* 68 9024–9032. 10.1158/0008-5472.CAN-08-2494 18974148 PMC2584362

[B11] Battaje R. R. Panda D. (2017). Lessons from bacterial homolog of tubulin, FtsZ for microtubule dynamics. *Endocr. Relat. Cancer* 24 T1–T21. 10.1530/ERC-17-0118 28634179

[B12] Beall B. Lutkenhaus J. (1991). FtsZ in *Bacillus subtilis* is required for vegetative septation and for asymmetric septation during sporulation. *Genes Dev.* 5 447–455. 10.1101/gad.5.3.447 1848202

[B13] Bergman P. Johansson L. Wan H. Jones A. Gallo R. L. Gudmundsson G. H. (2006). Induction of the antimicrobial peptide CRAMP in the blood-brain barrier and meninges after meningococcal infection. *Infect. Immun.* 74 6982–6991. 10.1128/IAI.01043-06 17030578 PMC1698100

[B14] Beuria T. K. Santra M. K. Panda D. (2005). Sanguinarine blocks cytokinesis in bacteria by inhibiting FtsZ assembly and bundling. *Biochemistry* 44 16584–16593. 16342949 10.1021/bi050767+

[B15] Beuria T. K. Singh P. Surolia A. Panda D. (2009). Promoting assembly and bundling of FtsZ as a strategy to inhibit bacterial cell division: a new approach for developing novel antibacterial drugs. *Biochem. J.* 423 61–69. 10.1042/BJ20090817 19583568

[B16] Bhattacharya A. Jindal B. Singh P. Datta A. Panda D. (2013). Plumbagin inhibits cytokinesis in *Bacillus subtilis* by inhibiting FtsZ assembly–a mechanistic study of its antibacterial activity. *FEBS J.* 280 4585–4599. 10.1111/febs.12429 23841620

[B17] Bi E. F. Lutkenhaus J. (1991). FtsZ ring structure associated with division in *Escherichia coli*. *Nature* 354 161–164. 10.1038/354161a0 1944597

[B18] Bi F. Guo L. Wang Y. Venter H. Semple S. J. Liu F. (2017). Design, synthesis and biological activity evaluation of novel 2,6-difluorobenzamide derivatives through FtsZ inhibition. *Bioorg. Med. Chem. Lett.* 27 958–962. 10.1016/j.bmcl.2016.12.081 28082038

[B19] Bi F. Song D. Qin Y. Liu X. Teng Y. Zhang N. (2019). Discovery of 1,3,4-oxadiazol-2-one-containing benzamide derivatives targeting FtsZ as highly potent agents of killing a variety of MDR bacteria strains. *Bioorg. Med. Chem.* 27 3179–3193. 10.1016/j.bmc.2019.06.010 31200986

[B20] Bi F. Song D. Zhang N. Liu Z. Gu X. Hu C. (2018). Design, synthesis and structure-based optimization of novel isoxazole-containing benzamide derivatives as FtsZ modulators. *Eur. J. Med. Chem.* 159 90–103. 10.1016/j.ejmech.2018.09.053 30268826

[B21] Bisson-Filho A. W. Discola K. F. Castellen P. Blasios V. Martins A. Sforca M. L. (2015). FtsZ filament capping by MciZ, a developmental regulator of bacterial division. *Proc. Natl. Acad. Sci. U.S.A.* 112 E2130–E2138. 10.1073/pnas.1414242112 25848052 PMC4418908

[B22] Bisson-Filho A. W. Hsu Y. P. Squyres G. R. Kuru E. Wu F. Jukes C. (2017). Treadmilling by FtsZ filaments drives peptidoglycan synthesis and bacterial cell division. *Science* 355 739–743. 10.1126/science.aak9973 28209898 PMC5485650

[B23] Borges A. Ferreira C. Saavedra M. J. Simoes M. (2013). Antibacterial activity and mode of action of ferulic and gallic acids against pathogenic bacteria. *Microb. Drug Resist.* 19 256–265. 10.1089/mdr.2012.0244 23480526

[B24] Bramhill D. Thompson C. M. (1994). GTP-dependent polymerization of *Escherichia coli* FtsZ protein to form tubules. *Proc. Natl. Acad. Sci. U.S.A.* 91 5813–5817. 10.1073/pnas.91.13.5813 8016071 PMC44087

[B25] Bramkamp M. Emmins R. Weston L. Donovan C. Daniel R. A. Errington J. (2008). A novel component of the division-site selection system of *Bacillus subtilis* and a new mode of action for the division inhibitor MinCD. *Mol. Microbiol.* 70 1556–1569. 10.1111/j.1365-2958.2008.06501.x 19019154

[B26] Buske P. J. Levin P. A. (2012). Extreme *C terminus* of bacterial cytoskeletal protein FtsZ plays fundamental role in assembly independent of modulatory proteins. *J. Biol. Chem.* 287 10945–10957. 10.1074/jbc.M111.330324 22298780 PMC3322825

[B27] Cai S. Yuan W. Li Y. Huang X. Guo Q. Tang Z. (2019). Antibacterial activity of indolyl-quinolinium derivatives and study their mode of action. *Bioorg. Med. Chem.* 27 1274–1282. 10.1016/j.bmc.2019.02.024 30792100

[B28] Carro L. (2019). Recent progress in the development of small-molecule FtsZ inhibitors as chemical tools for the development of novel antibiotics. *Antibiotics (Basel)* 8:217. 10.3390/antibiotics8040217 31717975 PMC6963470

[B29] Casiraghi A. Suigo L. Valoti E. Straniero V. (2020). Targeting bacterial cell division: a binding site-centered approach to the most promising inhibitors of the essential protein FtsZ. *Antibiotics (Basel)* 9:69. 10.3390/antibiotics9020069 32046082 PMC7167804

[B30] Chakraborti S. Das L. Kapoor N. Das A. Dwivedi V. Poddar A. (2011). Curcumin recognizes a unique binding site of tubulin. *J. Med. Chem.* 54 6183–6196. 10.1021/jm2004046 21830815

[B31] Chan F. Y. Sun N. Neves M. A. Lam P. C. Chung W. H. Wong L. K. (2013). Identification of a new class of FtsZ inhibitors by structure-based design and in vitro screening. *J. Chem. Inf. Model.* 53 2131–2140. 10.1021/ci400203f 23848971

[B32] Chang M. Y. Shen Y. L. (2014). Linalool exhibits cytotoxic effects by activating antitumor immunity. *Molecules* 19 6694–6706. 10.3390/molecules19056694 24858101 PMC6271996

[B33] Cohan M. C. Eddelbuettel A. M. P. Levin P. A. Pappu R. V. (2020). Dissecting the functional contributions of the intrinsically disordered C-terminal tail of *Bacillus subtilis* FtsZ. *J. Mol. Biol.* 432 3205–3221. 10.1016/j.jmb.2020.03.008 32198113 PMC8922553

[B34] Dai K. Lutkenhaus J. (1991). ftsZ is an essential cell division gene in *Escherichia coli*. *J. Bacteriol.* 173 3500–3506. 10.1128/jb.173.11.3500-3506.1991 2045370 PMC207964

[B35] de Boer P. Crossley R. Rothfield L. (1992). The essential bacterial cell-division protein FtsZ is a GTPase. *Nature* 359 254–256. 10.1038/359254a0 1528268

[B36] de Paiva S. R. Figueiredo M. R. Aragao T. V. Kaplan M. A. (2003). Antimicrobial activity in vitro of plumbagin isolated from *Plumbago* species. *Mem. Inst. Oswaldo. Cruz.* 98 959–961. 10.1590/s0074-02762003000700017 14762525

[B37] de Pereda J. M. Leynadier D. Evangelio J. A. Chacon P. Andreu J. M. (1996). Tubulin secondary structure analysis, limited proteolysis sites, and homology to FtsZ. *Biochemistry* 35 14203–14215. 10.1021/bi961357b 8916905

[B38] de Souza S. M. Delle Monache F. Smania A. Jr. (2005). Antibacterial activity of coumarins. *Z. Naturforsch. C. J. Biosci.* 60 693–700. 10.1515/znc-2005-9-1006 16320610

[B39] Detsi A. Kontogiorgis C. Hadjipavlou-Litina D. (2017). Coumarin derivatives: an updated patent review (2015-2016). *Expert Opin. Ther. Pat.* 27 1201–1226. 10.1080/13543776.2017.1360284 28756713

[B40] Dhaked H. P. Bhattacharya A. Yadav S. Dantu S. C. Kumar A. Panda D. (2016). Mutation of Arg191 in FtsZ impairs cytokinetic abscission of *Bacillus subtilis* cells. *Biochemistry* 55 5754–5763. 10.1021/acs.biochem.6b00493 27629358

[B41] Din N. Quardokus E. M. Sackett M. J. Brun Y. V. (1998). Dominant C-terminal deletions of FtsZ that affect its ability to localize in *Caulobacter* and its interaction with FtsA. *Mol. Microbiol.* 27 1051–1063. 10.1046/j.1365-2958.1998.00752.x 9535094

[B42] Domadia P. Swarup S. Bhunia A. Sivaraman J. Dasgupta D. (2007). Inhibition of bacterial cell division protein FtsZ by cinnamaldehyde. *Biochem. Pharmacol.* 74 831–840. 10.1016/j.bcp.2007.06.029 17662960

[B43] Domadia P. N. Bhunia A. Sivaraman J. Swarup S. Dasgupta D. (2008). Berberine targets assembly of *Escherichia coli* cell division protein FtsZ. *Biochemistry* 47 3225–3234. 10.1021/bi7018546 18275156

[B44] Duggirala S. Nankar R. P. Rajendran S. Doble M. (2014). Phytochemicals as inhibitors of bacterial cell division protein FtsZ: coumarins are promising candidates. *Appl. Biochem. Biotechnol.* 174 283–296. 10.1007/s12010-014-1056-2 25062781

[B45] Duman R. Ishikawa S. Celik I. Strahl H. Ogasawara N. Troc P. (2013). Structural and genetic analyses reveal the protein SepF as a new membrane anchor for the Z ring. *Proc. Natl. Acad. Sci. U.S.A.* 110 E4601–E4610. 10.1073/pnas.1313978110 24218584 PMC3845145

[B46] Elokdah H. Abou-Gharbia M. Hennan J. K. McFarlane G. Mugford C. P. Krishnamurthy G. (2004). Tiplaxtinin, a novel, orally efficacious inhibitor of plasminogen activator inhibitor-1: design, synthesis, and preclinical characterization. *J. Med. Chem.* 47 3491–3494. 10.1021/jm049766q 15214776

[B47] Erickson H. P. (1995). FtsZ, a prokaryotic homolog of tubulin? *Cell* 80 367–370. 10.1016/0092-8674(95)90486-7 7859278

[B48] Erickson H. P. (1998). Atomic structures of tubulin and FtsZ. *Trends Cell Biol.* 8 133–137. 10.1016/s0962-8924(98)01237-9 9695825

[B49] Erickson H. P. Anderson D. E. Osawa M. (2010). FtsZ in bacterial cytokinesis: cytoskeleton and force generator all in one. *Microbiol. Mol. Biol. Rev.* 74 504–528. 10.1128/MMBR.00021-10 21119015 PMC3008173

[B50] Eroglu C. Secme M. Bagci G. Dodurga Y. (2015). Assessment of the anticancer mechanism of ferulic acid via cell cycle and apoptotic pathways in human prostate cancer cell lines. *Tumour. Biol.* 36 9437–9446. 10.1007/s13277-015-3689-3 26124008

[B51] Fang Z. Zheng S. Chan K. F. Yuan W. Guo Q. Wu W. (2019). Design, synthesis and antibacterial evaluation of 2,4-disubstituted-6-thiophenyl-pyrimidines. *Eur. J. Med. Chem.* 161 141–153. 10.1016/j.ejmech.2018.10.039 30347327

[B52] Ferrer-Gonzalez E. Fujita J. Yoshizawa T. Nelson J. M. Pilch A. J. Hillman E. (2019). Structure-guided design of a fluorescent probe for the visualization of FtsZ in clinically important gram-positive and gram-negative bacterial pathogens. *Sci. Rep.* 9:20092. 10.1038/s41598-019-56557-x 31882782 PMC6934700

[B53] Finn G. J. Creaven B. Egan D. A. (2001). Study of the in vitro cytotoxic potential of natural and synthetic coumarin derivatives using human normal and neoplastic skin cell lines. *Melanoma Res.* 11 461–467. 10.1097/00008390-200110000-00004 11595882

[B54] Fujita J. Maeda Y. Mizohata E. Inoue T. Kaul M. Parhi A. K. (2017). Structural flexibility of an inhibitor overcomes drug resistance mutations in *Staphylococcus aureus* FtsZ. *ACS Chem. Biol.* 12 1947–1955. 10.1021/acschembio.7b00323 28621933 PMC5705026

[B55] Gardner K. A. Moore D. A. Erickson H. P. (2013). The C-terminal linker of *Escherichia coli* FtsZ functions as an intrinsically disordered peptide. *Mol. Microbiol.* 89 264–275. 10.1111/mmi.12279 23714328 PMC3725778

[B56] Guo Z. Li B. Cheng L. T. Zhou S. McCammon J. A. Che J. (2015). Identification of protein-ligand binding sites by the level-set variational implicit-solvent approach. *J. Chem. Theory Comput.* 11 753–765. 10.1021/ct500867u 25941465 PMC4410907

[B57] Haeusser D. P. Hoashi M. Weaver A. Brown N. Pan J. Sawitzke J. A. (2014). The Kil peptide of bacteriophage lambda blocks *Escherichia coli* cytokinesis via ZipA-dependent inhibition of FtsZ assembly. *PLoS Genet.* 10:e1004217. 10.1371/journal.pgen.1004217 24651041 PMC3961180

[B58] Haeusser D. P. Margolin W. (2016). Splitsville: structural and functional insights into the dynamic bacterial Z ring. *Nat. Rev. Microbiol.* 14 305–319. 27040757 10.1038/nrmicro.2016.26 PMC5290750

[B59] Haeusser D. P. Schwartz R. L. Smith A. M. Oates M. E. Levin P. A. (2004). EzrA prevents aberrant cell division by modulating assembly of the cytoskeletal protein FtsZ. *Mol. Microbiol.* 52 801–814. 10.1111/j.1365-2958.2004.04016.x 15101985 PMC5517308

[B60] Hale C. A. de Boer P. A. (1997). Direct binding of FtsZ to ZipA, an essential component of the septal ring structure that mediates cell division in *E. coli*. *Cell* 88 175–185. 10.1016/s0092-8674(00)81838-3 9008158

[B61] Handler A. A. Lim J. E. Losick R. (2008). Peptide inhibitor of cytokinesis during sporulation in *Bacillus subtilis*. *Mol. Microbiol.* 68 588–599. 10.1111/j.1365-2958.2008.06173.x 18284588 PMC2603569

[B62] Haranahalli K. Tong S. Ojima I. (2016). Recent advances in the discovery and development of antibacterial agents targeting the cell-division protein FtsZ. *Bioorg. Med. Chem.* 24 6354–6369. 10.1016/j.bmc.2016.05.003 27189886 PMC5157688

[B63] Haydon D. J. Stokes N. R. Ure R. Galbraith G. Bennett J. M. Brown D. R. (2008). An inhibitor of FtsZ with potent and selective anti-staphylococcal activity. *Science* 321 1673–1675. 10.1126/science.1159961 18801997

[B64] Hemaiswarya S. Doble M. (2009). Synergistic interaction of eugenol with antibiotics against Gram negative bacteria. *Phytomedicine* 16 997–1005. 10.1016/j.phymed.2009.04.006 19540744

[B65] Hemaiswarya S. Doble M. (2010). Synergistic interaction of phenylpropanoids with antibiotics against bacteria. *J. Med. Microbiol.* 59(Pt 12) 1469–1476. 10.1099/jmm.0.022426-0 20724513

[B66] Hemaiswarya S. Soudaminikkutty R. Narasumani M. L. Doble M. (2011). Phenylpropanoids inhibit protofilament formation of *Escherichia coli* cell division protein FtsZ. *J. Med. Microbiol.* 60(Pt 9) 1317–1325. 10.1099/jmm.0.030536-0 21474608

[B67] Hernandez-Rocamora V. M. Alfonso C. Margolin W. Zorrilla S. Rivas G. (2015). Evidence that bacteriophage lambda kil peptide inhibits bacterial cell division by disrupting FtsZ protofilaments and sequestering protein subunits. *J. Biol. Chem.* 290 20325–20335. 10.1074/jbc.M115.653329 26124275 PMC4536439

[B68] Hsin J. Gopinathan A. Huang K. C. (2012). Nucleotide-dependent conformations of FtsZ dimers and force generation observed through molecular dynamics simulations. *Proc. Natl. Acad. Sci. U.S.A.* 109 9432–9437. 10.1073/pnas.1120761109 22647609 PMC3386107

[B69] Huang Q. Kirikae F. Kirikae T. Pepe A. Amin A. Respicio L. (2006). Targeting FtsZ for antituberculosis drug discovery: noncytotoxic taxanes as novel antituberculosis agents. *J. Med. Chem.* 49 463–466. 10.1021/jm050920y 16420032 PMC2527599

[B70] Huecas S. Araujo-Bazan L. Ruiz F. M. Ruiz-Avila L. B. Martinez R. F. Escobar-Pena A. (2021). Targeting the FtsZ allosteric binding site with a novel fluorescence polarization screen, cytological and structural approaches for antibacterial discovery. *J. Med. Chem.* 64 5730–5745. 10.1021/acs.jmedchem.0c02207 33908781 PMC8478281

[B71] Jaiswal R. Beuria T. K. Mohan R. Mahajan S. K. Panda D. (2007). Totarol inhibits bacterial cytokinesis by perturbing the assembly dynamics of FtsZ. *Biochemistry* 46 4211–4220. 10.1021/bi602573e 17348691

[B72] Jindal B. Panda D. (2013). Understanding FtsZ assembly: cues from the behavior of its N- and C-terminal domains. *Biochemistry* 52 7071–7081. 10.1021/bi400129j 24007276

[B73] Kaul M. Mark L. Zhang Y. Parhi A. K. LaVoie E. J. Pilch D. S. (2013). Pharmacokinetics and in vivo antistaphylococcal efficacy of TXY541, a 1-methylpiperidine-4-carboxamide prodrug of PC190723. *Biochem. Pharmacol.* 86 1699–1707. 10.1016/j.bcp.2013.10.010 24148278

[B74] Kaul M. Mark L. Zhang Y. Parhi A. K. Lyu Y. L. Pawlak J. (2015). TXA709, an FtsZ-targeting benzamide prodrug with improved pharmacokinetics and enhanced in vivo efficacy against methicillin-resistant *Staphylococcus aureus*. *Antimicrob. Agents Chemother.* 59 4845–4855. 10.1128/AAC.00708-15 26033735 PMC4505295

[B75] Kaul M. Zhang Y. Parhi A. K. Lavoie E. J. Pilch D. S. (2014). Inhibition of RND-type efflux pumps confers the FtsZ-directed prodrug TXY436 with activity against Gram-negative bacteria. *Biochem. Pharmacol.* 89 321–328. 10.1016/j.bcp.2014.03.002 24637241

[B76] Kaur S. Modi N. H. Panda D. Roy N. (2010). Probing the binding site of curcumin in *Escherichia coli* and *Bacillus subtilis* FtsZ–a structural insight to unveil antibacterial activity of curcumin. *Eur. J. Med. Chem.* 45 4209–4214. 10.1016/j.ejmech.2010.06.015 20615583

[B77] Keffer J. L. Huecas S. Hammill J. T. Wipf P. Andreu J. M. Bewley C. A. (2013). Chrysophaentins are competitive inhibitors of FtsZ and inhibit Z-ring formation in live bacteria. *Bioorg. Med. Chem.* 21 5673–5678. 10.1016/j.bmc.2013.07.033 23932448 PMC3768135

[B78] Kellogg E. H. Hejab N. M. A. Howes S. Northcote P. Miller J. H. Diaz J. F. (2017). Insights into the distinct mechanisms of action of taxane and non-taxane microtubule stabilizers from cryo-EM structures. *J. Mol. Biol.* 429 633–646. 10.1016/j.jmb.2017.01.001 28104363 PMC5325780

[B79] Keri R. S. Sasidhar B. S. Nagaraja B. M. Santos M. A. (2015). Recent progress in the drug development of coumarin derivatives as potent antituberculosis agents. *Eur. J. Med. Chem.* 100 257–269. 10.1016/j.ejmech.2015.06.017 26112067

[B80] Khare S. Hsin J. Sorto N. A. Nepomuceno G. M. Shaw J. T. Shi H. (2019). FtsZ-Independent mechanism of division inhibition by the small molecule PC190723 in *Escherichia coli*. *Adv. Biosyst.* 3:e1900021. 10.1002/adbi.201900021 32648693

[B81] Kiro R. Molshanski-Mor S. Yosef I. Milam S. L. Erickson H. P. Qimron U. (2013). Gene product 0.4 increases bacteriophage T7 competitiveness by inhibiting host cell division. *Proc. Natl. Acad. Sci. U.S.A.* 110 19549–19554. 10.1073/pnas.1314096110 24218612 PMC3845191

[B82] Krupka M. Margolin W. (2018). Unite to divide: oligomerization of tubulin and actin homologs regulates initiation of bacterial cell division. *F1000Res.* 7:235. 10.12688/f1000research.13504.1 PMC5832921 29560258

[B83] Kusuma K. D. Griffith R. Harry E. J. Bottomley A. L. Ung A. T. (2019). In silico analysis of FtsZ crystal structures towards a new target for antibiotics. *Aust. J. Chem.* 72 184–193. 10.1071/CH18347

[B84] Lan G. Wolgemuth C. W. Sun S. X. (2007). Z-ring force and cell shape during division in rod-like bacteria. *Proc. Natl. Acad. Sci. U.S.A.* 104 16110–16115. 10.1073/pnas.0702925104 17913889 PMC2042170

[B85] Li X. Ma S. (2015). Advances in the discovery of novel antimicrobials targeting the assembly of bacterial cell division protein FtsZ. *Eur. J. Med. Chem.* 95 1–15. 10.1016/j.ejmech.2015.03.026 25791674

[B86] Li Y. Sun N. Ser H.-L. Long W. Li Y. Chen C. (2015). Antibacterial activity evaluation and mode of action study of novel thiazole-quinolinium derivatives. *RSC Adv.* 10 15000–15014. 10.1039/D0RA00691B PMC9052103 35497125

[B87] Lou Z. Wang H. Rao S. Sun J. Chaoyang m Li J. (2012). P-Coumaric acid kills bacteria through dual damage mechanisms. *Food Control* 25 550–554. 10.1016/j.foodcont.2011.11.022

[B88] Löwe J. (1998). Crystal structure determination of FtsZ from *Methanococcus jannaschii*. *J. Struct. Biol.* 124 235–243. 10.1006/jsbi.1998.4041 10049809

[B89] Löwe J. Amos L. A. (1998). Crystal structure of the bacterial cell-division protein FtsZ. *Nature* 391 203–206. 10.1038/34472 9428770

[B90] Löwe J. Amos L. A. (1999). Tubulin-like protofilaments in Ca2+-induced FtsZ sheets. *EMBO J.* 18 2364–2371. 10.1093/emboj/18.9.2364 10228151 PMC1171319

[B91] Lu C. Reedy M. Erickson H. P. (2000). Straight and curved conformations of FtsZ are regulated by GTP hydrolysis. *J. Bacteriol.* 182 164–170. 10.1128/JB.182.1.164-170.2000 10613876 PMC94253

[B92] Lu C. Stricker J. Erickson H. P. (1998). FtsZ from *Escherichia coli*, *Azotobacter vinelandii*, and *Thermotoga maritima*–quantitation, GTP hydrolysis, and assembly. *Cell Motil. Cytoskeleton* 40 71–86. 10.1002/(SICI)1097-0169199840:1<71::AID-CM7<3.0.CO;2-I 9605973

[B93] Lu Y. Chen J. Xiao M. Li W. Miller D. D. (2012). An overview of tubulin inhibitors that interact with the colchicine binding site. *Pharm. Res.* 29 2943–2971. 10.1007/s11095-012-0828-z 22814904 PMC3667160

[B94] Lui H. K. Gao W. Cheung K. C. Jin W. B. Sun N. Kan J. W. Y. (2019). Boosting the efficacy of anti-MRSA beta-lactam antibiotics via an easily accessible, non-cytotoxic and orally bioavailable FtsZ inhibitor. *Eur. J. Med. Chem.* 163 95–115. 10.1016/j.ejmech.2018.11.052 30503946

[B95] Ma S. Ma S. (2012). The development of FtsZ inhibitors as potential antibacterial agents. *ChemMedChem* 7 1161–1172. 10.1002/cmdc.201200156 22639193

[B96] Ma X. Margolin W. (1999). Genetic and functional analyses of the conserved C-terminal core domain of *Escherichia coli* FtsZ. *J. Bacteriol.* 181 7531–7544. 10601211 10.1128/jb.181.24.7531-7544.1999 PMC94211

[B97] Mathew B. Hobrath J. V. Ross L. Connelly M. C. Lofton H. Rajagopalan M. (2016). Screening and development of new inhibitors of FtsZ from *M. Tuberculosis*. *PLoS One* 11:e0164100. 10.1371/journal.pone.0164100 27768711 PMC5074515

[B98] Mathew B. Ross L. Reynolds R. C. (2013). A novel quinoline derivative that inhibits mycobacterial FtsZ. *Tuberculosis (Edinb.)* 93 398–400. 10.1016/j.tube.2013.04.002 23647650 PMC3686551

[B99] Mathew R. Kruthiventi A. K. Prasad J. V. Kumar S. P. Srinu G. Chatterji D. (2010). Inhibition of mycobacterial growth by plumbagin derivatives. *Chem. Biol. Drug Des.* 76 34–42. 10.1111/j.1747-0285.2010.00987.x 20456370

[B100] Matsui T. Lallo S. Nisa K. Morita H. (2017). Filamenting temperature-sensitive mutant Z inhibitors from *Glycyrrhiza glabra* and their inhibitory mode of action. *Bioorg. Med. Chem. Lett.* 27 1420–1424. 10.1016/j.bmcl.2017.01.095 28196701

[B101] Matsui T. Yamane J. Mogi N. Yamaguchi H. Takemoto H. Yao M. (2012). Structural reorganization of the bacterial cell-division protein FtsZ from *Staphylococcus aureus*. *Acta Crystallogr. D Biol. Crystallogr.* 68(Pt 9) 1175–1188. 10.1107/S0907444912022640 22948918

[B102] Miguel A. Hsin J. Liu T. Tang G. Altman R. B. Huang K. C. (2015). Variations in the binding pocket of an inhibitor of the bacterial division protein FtsZ across genotypes and species. *PLoS Comput. Biol.* 11:e1004117. 10.1371/journal.pcbi.1004117 25811761 PMC4374959

[B103] Mukherjee A. Dai K. Lutkenhaus J. (1993). *Escherichia coli* cell division protein FtsZ is a guanine nucleotide binding protein. *Proc. Natl. Acad. Sci. U.S.A.* 90 1053–1057. 10.1073/pnas.90.3.1053 8430073 PMC45809

[B104] Mukherjee A. Lutkenhaus J. (1994). Guanine nucleotide-dependent assembly of FtsZ into filaments. *J. Bacteriol.* 176 2754–2758. 10.1128/jb.176.9.2754-2758.1994 8169229 PMC205420

[B105] Ng L. T. Wu S. J. (2011). Antiproliferative activity of *Cinnamomum cassia* constituents and effects of pifithrin-alpha on their apoptotic signaling pathways in Hep G2 Cells. *Evid. Based Complement Alternat. Med.* 2011:492148. 10.1093/ecam/nep220 20038571 PMC3135661

[B106] Nguyen L. T. Oikonomou C. M. Ding H. J. Kaplan M. Yao Q. Chang Y. W. (2019). Simulations suggest a constrictive force is required for Gram-negative bacterial cell division. *Nat. Commun.* 10:1259. 10.1038/s41467-019-09264-0 30890709 PMC6425016

[B107] Niero E. L. Machado-Santelli G. M. (2013). Cinnamic acid induces apoptotic cell death and cytoskeleton disruption in human melanoma cells. *J. Exp. Clin. Cancer Res.* 32:31. 10.1186/1756-9966-32-31 23701745 PMC3667113

[B108] Nogales E. Downing K. H. Amos L. A. Löwe J. (1998a). Tubulin and FtsZ form a distinct family of GTPases. *Nat. Struct. Biol.* 5 451–458. 10.1038/nsb0698-451 9628483

[B109] Nogales E. Wolf S. G. Downing K. H. (1998b). Structure of the alpha beta tubulin dimer by electron crystallography. *Nature* 391 199–203. 10.1038/34465 9428769

[B110] Nussbaum P. Gerstner M. Dingethal M. Erb C. Albers S. V. (2021). The archaeal protein SepF is essential for cell division in *Haloferax volcanii*. *Nat. Commun.* 12:3469. 10.1038/s41467-021-23686-9 34103513 PMC8187382

[B111] Ohashi Y. Chijiiwa Y. Suzuki K. Takahashi K. Nanamiya H. Sato T. (1999). The lethal effect of a benzamide derivative, 3-methoxybenzamide, can be suppressed by mutations within a cell division gene, ftsZ, in *Bacillus subtilis*. *J. Bacteriol.* 181 1348–1351. 10.1128/JB.181.4.1348-1351.1999 9973366 PMC93517

[B112] Oncul S. Ercan A. (2017). Discrimination of the effects of doxorubicin on two different breast cancer cell lines on account of multidrug resistance and apoptosis. *Indian J. Pharm. Sci.* 79 599–607. 10.4172/pharmaceutical-sciences.1000268

[B113] Ortiz C. Natale P. Cueto L. Vicente M. (2016). The keepers of the ring: regulators of FtsZ assembly. *FEMS Microbiol. Rev.* 40 57–67. 26377318 10.1093/femsre/fuv040

[B114] Panda D. Bhattacharya D. Gao Q. H. Oza P. M. Lin H. Y. Hawkins B. (2016). Identification of agents targeting FtsZ assembly. *Future Med. Chem.* 8 1111–1132. 10.4155/fmc-2016-0041 27284850

[B115] Panda P. Taviti A. C. Satpati S. Kar M. M. Dixit A. Beuria T. K. (2015). Doxorubicin inhibits *E. coli* division by interacting at a novel site in FtsZ. *Biochem. J.* 471 335–346. 10.1042/BJ20150467 26285656

[B116] Pichoff S. Lutkenhaus J. (2002). Unique and overlapping roles for ZipA and FtsA in septal ring assembly in *Escherichia coli*. *EMBO J.* 21 685–693. 10.1093/emboj/21.4.685 11847116 PMC125861

[B117] Piddock L. J. Walters R. N. (1992). Bactericidal activities of five quinolones for *Escherichia coli* strains with mutations in genes encoding the SOS response or cell division. *Antimicrob. Agents Chemother.* 36 819–825. 10.1128/aac.36.4.819 1503444 PMC189433

[B118] Pinho M. G. Errington J. (2003). Dispersed mode of *Staphylococcus aureus* cell wall synthesis in the absence of the division machinery. *Mol. Microbiol.* 50 871–881. 10.1046/j.1365-2958.2003.03719.x 14617148

[B119] Plaza A. Keffer J. L. Bifulco G. Lloyd J. R. Bewley C. A. (2010). Chrysophaentins A-H, antibacterial bisdiarylbutene macrocycles that inhibit the bacterial cell division protein FtsZ. *J. Am. Chem. Soc.* 132 9069–9077. 10.1021/ja102100h 20536175 PMC2920594

[B120] Prota A. E. Bargsten K. Northcote P. T. Marsh M. Altmann K. H. Miller J. H. (2014). Structural basis of microtubule stabilization by laulimalide and peloruside A. *Angew Chem. Int. Ed. Engl.* 53 1621–1625. 10.1002/anie.201307749 24470331

[B121] Puupponen-Pimia R. Nohynek L. Meier C. Kahkonen M. Heinonen M. Hopia A. (2001). Antimicrobial properties of phenolic compounds from berries. *J. Appl. Microbiol.* 90 494–507. 10.1046/j.1365-2672.2001.01271.x 11309059

[B122] Raghav D. Ashraf S. M. Mohan L. Rathinasamy K. (2017). Berberine induces toxicity in HeLa cells through perturbation of microtubule polymerization by binding to tubulin at a unique site. *Biochemistry* 56 2594–2611. 10.1021/acs.biochem.7b00101 28459539

[B123] Rai D. Singh J. K. Roy N. Panda D. (2008). Curcumin inhibits FtsZ assembly: an attractive mechanism for its antibacterial activity. *Biochem. J.* 410 147–155. 10.1042/BJ20070891 17953519

[B124] Ramirez-Diaz D. A. Merino-Salomon A. Meyer F. Heymann M. Rivas G. Bramkamp M. (2021). FtsZ induces membrane deformations via torsional stress upon GTP hydrolysis. *Nat. Commun.* 12:3310. 10.1038/s41467-021-23387-3 34083531 PMC8175707

[B125] Rastogi N. Domadia P. Shetty S. Dasgupta D. (2008). Screening of natural phenolic compounds for potential to inhibit bacterial cell division protein FtsZ. *Indian J. Exp. Biol.* 46 783–787. 19090350

[B126] Ravelli R. B. Gigant B. Curmi P. A. Jourdain I. Lachkar S. Sobel A. (2004). Insight into tubulin regulation from a complex with colchicine and a stathmin-like domain. *Nature* 428 198–202. 10.1038/nature02393 15014504

[B127] Ray S. Dhaked H. P. Panda D. (2014). Antimicrobial peptide CRAMP (16-33) stalls bacterial cytokinesis by inhibiting FtsZ assembly. *Biochemistry* 53 6426–6429. 10.1021/bi501115p 25294259

[B128] Ray S. Jindal B. Kunal K. Surolia A. Panda D. (2015). BT-benzo-29 inhibits bacterial cell proliferation by perturbing FtsZ assembly. *FEBS J.* 282 4015–4033. 10.1111/febs.13403 26258635

[B129] Ray S. Kumar A. Panda D. (2013). GTP regulates the interaction between MciZ and FtsZ: a possible role of MciZ in bacterial cell division. *Biochemistry* 52 392–401. 10.1021/bi301237m 23237472

[B130] Romberg L. Simon M. Erickson H. P. (2001). Polymerization of Ftsz, a bacterial homolog of tubulin. is assembly cooperative? *J. Biol. Chem.* 276 11743–11753. 10.1074/jbc.M009033200 11152458

[B131] Rowlett V. W. Margolin W. (2015). The Min system and other nucleoid-independent regulators of Z ring positioning. *Front. Microbiol.* 6:478. 10.3389/fmicb.2015.00478 26029202 PMC4429545

[B132] Ruiz-Avila L. B. Huecas S. Artola M. Vergonos A. Ramirez-Aportela E. Cercenado E. (2013). Synthetic inhibitors of bacterial cell division targeting the GTP-binding site of FtsZ. *ACS Chem. Biol.* 8 2072–2083. 10.1021/cb400208z 23855511

[B133] Sanderson J. T. Clabault H. Patton C. Lassalle-Claux G. Jean-Francois J. Pare A. F. (2013). Antiproliferative, antiandrogenic and cytotoxic effects of novel caffeic acid derivatives in LNCaP human androgen-dependent prostate cancer cells. *Bioorg. Med. Chem.* 21 7182–7193. 10.1016/j.bmc.2013.08.057 24080105

[B134] Santra M. K. Beuria T. K. Banerjee A. Panda D. (2004). Ruthenium red-induced bundling of bacterial cell division protein, FtsZ. *J. Biol. Chem.* 279 25959–25965. 10.1074/jbc.M312473200 15039432

[B135] Scheffers D. J. de Wit J. G. den Blaauwen T. Driessen A. J. (2002). GTP hydrolysis of cell division protein FtsZ: evidence that the active site is formed by the association of monomers. *Biochemistry* 41 521–529. 10.1021/bi011370i 11781090

[B136] Schumacher M. A. Ohashi T. Corbin L. Erickson H. P. (2020). High-resolution crystal structures of *Escherichia coli* FtsZ bound to GDP and GTP. *Acta Crystallogr. F Struct. Biol. Commun.* 76(Pt 2) 94–102. 10.1107/S2053230X20001132 32039891 PMC7010359

[B137] Singh D. Bhattacharya A. Rai A. Dhaked H. P. Awasthi D. Ojima I. (2014). SB-RA-2001 inhibits bacterial proliferation by targeting FtsZ assembly. *Biochemistry* 53 2979–2992. 10.1021/bi401356y 24749867 PMC4020581

[B138] Singh P. Jindal B. Surolia A. Panda D. (2012). A rhodanine derivative CCR-11 inhibits bacterial proliferation by inhibiting the assembly and GTPase activity of FtsZ. *Biochemistry* 51 5434–5442. 10.1021/bi201813u 22703373

[B139] Squyres G. R. Holmes M. J. Barger S. R. Pennycook B. R. Ryan J. Yan V. T. (2021). Single-molecule imaging reveals that Z-ring condensation is essential for cell division in *Bacillus subtilis*. *Nat. Microbiol.* 6 553–562. 33737746 10.1038/s41564-021-00878-z PMC8085161

[B140] Sridevi D. Sudhakar K. U. Ananthathatmula R. Nankar R. P. Doble M. (2017). Mutation at G103 of MtbFtsZ altered their sensitivity to coumarins. *Front. Microbiol.* 8:578. 10.3389/fmicb.2017.00578 28428773 PMC5382161

[B141] Steinmetz M. O. Prota A. E. (2018). Microtubule-targeting agents: strategies to hijack the cytoskeleton. *Trends Cell Biol.* 28 776–792. 10.1016/j.tcb.2018.05.001 29871823

[B142] Straniero V. Sebastian-Perez V. Suigo L. Margolin W. Casiraghi A. Hrast M. (2021). Computational design and development of benzodioxane-benzamides as potent inhibitors of FtsZ by exploring the hydrophobic subpocket. *Antibiotics (Basel)* 10:442. 10.3390/antibiotics10040442 33920895 PMC8071314

[B143] Stricker J. Maddox P. Salmon E. D. Erickson H. P. (2002). Rapid assembly dynamics of the *Escherichia coli* FtsZ-ring demonstrated by fluorescence recovery after photobleaching. *Proc. Natl. Acad. Sci. U.S.A.* 99 3171–3175. 10.1073/pnas.052595099 11854462 PMC122491

[B144] Sun N. Chan F. Y. Lu Y. J. Neves M. A. Lui H. K. Wang Y. (2014). Rational design of berberine-based FtsZ inhibitors with broad-spectrum antibacterial activity. *PLoS One* 9:e97514. 10.1371/journal.pone.0097514 24824618 PMC4019636

[B145] Sun N. Du R. L. Zheng Y. Y. Guo Q. Cai S. Y. Liu Z. H. (2018). Antibacterial activity of 3-methylbenzo[d]thiazol-methylquinolinium derivatives and study of their action mechanism. *J. Enzyme Inhib. Med. Chem.* 33 879–889. 10.1080/14756366.2018.1465055 29722581 PMC6010097

[B146] Sun N. Du R. L. Zheng Y. Y. Huang B. H. Guo Q. Zhang R. F. (2017a). Antibacterial activity of N-methylbenzofuro[3,2-b]quinoline and N-methylbenzoindolo[3,2-b]-quinoline derivatives and study of their mode of action. *Eur. J. Med. Chem.* 135 1–11. 10.1016/j.ejmech.2017.04.018 28426995

[B147] Sun N. Lu Y. J. Chan F. Y. Du R. L. Zheng Y. Y. Zhang K. (2017b). A thiazole orange derivative targeting the bacterial protein FtsZ shows potent antibacterial activity. *Front. Microbiol.* 8:855. 10.3389/fmicb.2017.00855 28553278 PMC5426085

[B148] Sun N. Zheng Y. Y. Du R. L. Cai S. Y. Zhang K. So L. Y. (2017c). New application of tiplaxtinin as an effective FtsZ-targeting chemotype for an antimicrobial study. *Medchemcomm* 8 1909–1913. 10.1039/c7md00387k 30108711 PMC6072346

[B149] Taviti A. C. Beuria T. K. (2017). MinD directly interacting with FtsZ at the H10 helix suggests a model for robust activation of MinC to destabilize FtsZ polymers. *Biochem. J.* 474 3189–3205. 10.1042/BCJ20170357 28743721

[B150] Tripathy S. Sahu S. K. (2019). FtsZ inhibitors as a new genera of antibacterial agents. *Bioorg. Chem.* 91:103169. 10.1016/j.bioorg.2019.103169 31398602

[B151] Trusca D. Scott S. Thompson C. Bramhill D. (1998). Bacterial SOS checkpoint protein SulA inhibits polymerization of purified FtsZ cell division protein. *J. Bacteriol.* 180 3946–3953. 10.1128/JB.180.15.3946-3953.1998 9683493 PMC107380

[B152] Urgaonkar S. La Pierre H. S. Meir I. Lund H. RayChaudhuri D. Shaw J. T. (2005). Synthesis of antimicrobial natural products targeting FtsZ: (+/−)-dichamanetin and (+/−)-2′′′-hydroxy-5′′-benzylisouvarinol-B. *Org. Lett.* 7 5609–5612. 10.1021/ol052269z 16321003 PMC2588422

[B153] van den Ent F. Amos L. Löwe J. (2001). Bacterial ancestry of actin and tubulin. *Curr. Opin. Microbiol.* 4 634–638. 10.1016/s1369-5274(01)00262-4 11731313

[B154] Vaughan S. Wickstead B. Gull K. Addinall S. G. (2004). Molecular evolution of FtsZ protein sequences encoded within the genomes of archaea, bacteria, and eukaryota. *J. Mol. Evol.* 58 19–29. 10.1007/s00239-003-2523-5 14743312

[B155] Wagstaff J. M. Tsim M. Oliva M. A. Garcia-Sanchez A. Kureisaite-Ciziene D. Andreu J. M. (2017). A polymerization-associated structural switch in FtsZ that enables treadmilling of model filaments. *mBio* 8:e00254-17. 10.1128/mBio.00254-17 28465423 PMC5414002

[B156] Wang M. Fang C. Ma B. Luo X. Hou Z. (2020). Regulation of cytokinesis: FtsZ and its accessory proteins. *Curr. Genet.* 66 43–49. 10.1007/s00294-019-01005-6 31209564

[B157] White E. L. Suling W. J. Ross L. J. Seitz L. E. Reynolds R. C. (2002). 2-Alkoxycarbonylaminopyridines: inhibitors of *Mycobacterium tuberculosis* FtsZ. *J. Antimicrob. Chemother.* 50 111–114. 10.1093/jac/dkf075 12096015

[B158] White M. L. Eswara P. J. (2021). ylm has more than a (Z Anchor) ring to It! *J. Bacteriol.* 203:e00460-20. 10.1128/JB.00460-20 32900832 PMC7811201

[B159] Whitley K. D. Jukes C. Tregidgo N. Karinou E. Almada P. Cesbron Y. (2021). FtsZ treadmilling is essential for Z-ring condensation and septal constriction initiation in *Bacillus subtilis*. *Nat. Commun.* 12:2448. 10.1038/s41467-021-22526-0 PMC8079713 33907196

[B160] Wu Y. Y. Zhang T. Y. Zhang M. Y. Cheng J. Zhang Y. X. (2018). An endophytic Fungi of *Ginkgo biloba* L. produces antimicrobial metabolites as potential inhibitors of FtsZ of *Staphylococcus aureus*. *Fitoterapia* 128 265–271. 10.1016/j.fitote.2018.05.033 29864480

[B161] Yang X. Lyu Z. Miguel A. McQuillen R. Huang K. C. Xiao J. (2017). GTPase activity-coupled treadmilling of the bacterial tubulin FtsZ organizes septal cell wall synthesis. *Science* 355 744–747. 10.1126/science.aak9995 28209899 PMC5851775

[B162] Yao Q. Jewett A. I. Chang Y. W. Oikonomou C. M. Beeby M. Iancu C. V. (2017). Short FtsZ filaments can drive asymmetric cell envelope constriction at the onset of bacterial cytokinesis. *EMBO J.* 36 1577–1589. 10.15252/embj.201696235 28438890 PMC5452018

[B163] Yu H. H. Kim K. J. Cha J. D. Kim H. K. Lee Y. E. Choi N. Y. (2005). Antimicrobial activity of berberine alone and in combination with ampicillin or oxacillin against methicillin-resistant *Staphylococcus aureus*. *J. Med. Food* 8 454–461. 10.1089/jmf.2005.8.454 16379555

[B164] Zhang G. F. Zhang S. Pan B. Liu X. Feng L. S. (2018). 4-Quinolone derivatives and their activities against Gram positive pathogens. *Eur. J. Med. Chem.* 143 710–723. 10.1016/j.ejmech.2017.11.082 29220792

[B165] Zheng Y. Y. Du R. L. Cai S. Y. Liu Z. H. Fang Z. Y. Liu T. (2018). Study of benzofuroquinolinium derivatives as a new class of potent antibacterial agent and the mode of inhibition targeting FtsZ. *Front. Microbiol.* 9:1937. 10.3389/fmicb.2018.01937 30174667 PMC6107709

